# Street-Scale Nonlinear Associations Between 2D and 3D Plant Morphology and Land Surface Temperature

**DOI:** 10.3390/plants15172561

**Published:** 2026-08-23

**Authors:** Yufei Zhang, Shenghua Zhang, Yangyang Xu, Ming Chen, Yunxiao Guan

**Affiliations:** 1 FAFU-Dal Joint College, Fujian Agriculture and Forestry University, Fuzhou 350002, China; 3246804018@stu.fafu.edu.cn; 2 College of Landscape Architecture and Art, Fujian Agriculture and Forestry University, Fuzhou 350002, China; 52519052070@fafu.edu.cn (S.Z.); 52519052118@fafu.edu.cn (Y.X.)

**Keywords:** green view index, canopy structure, random forest, SHAP, spatial cross-validation, urban heat mitigation

## Abstract

Urban streets are important heat-exposure environments, yet the relationships of two-dimensional (2D) planar plant morphology and three-dimensional (3D) vegetation structure with land surface temperature (LST) remain insufficiently integrated at a continuous street scale. We analyzed 42,603 street-scale study units in the central urban area of Wuhan, defined at 50 m sampling intervals with a 150 m radius. The 2D variables comprised green-space area (A), mean patch perimeter (P_mean), and perimeter–area ratio (P_A), while the 3D variables comprised green view index (GVI), mean 3D green volume (NV_mean), mean canopy height (CH_mean), and canopy-height variability (CH_sd). Anselin Local Moran’s I identified High–High (HH) and Low–Low (LL) zones; linear regression (LR), random forest (RF), and SHAP characterized linear, nonlinear, and model-based contributions; and buffered spatial cross-validation and spatial resampling evaluated robustness. Under the original random 80–20% train–test split, LR/RF R^2^ values were 0.2153/0.4002 for the overall study area, 0.1940/0.3539 for the HH zone, and 0.0488/0.4853 for the LL zone. Under five-fold buffered spatial cross-validation, the corresponding pooled out-of-fold R^2^ values were 0.1940/0.2237, 0.1427/0.0965, and −0.0942/−0.0231, showing that the RF advantage weakened after spatial separation and did not persist in the HH and LL zones. In the original fitted RF models, A, P_mean, and NV_mean had the largest mean absolute SHAP contributions overall; A, P_A, and P_mean ranked highest in the HH zone; and GVI, CH_sd, and CH_mean ranked highest in the LL zone. Repeated buffered spatial validation showed no stable overall 2D predominance because the median 2D share of 52.1% had a 46.5–58.8% percentile range, but it supported stable 2D relative predominance in the HH zone (61.0% [55.5–66.7%]) and stable grouped 3D relative predominance in the LL zone (61.9% [52.3–70.3%]), despite unstable LL variable-level rankings. SHAP relationships were nonlinear and zone-dependent: A and P_mean showed clearer directional transitions overall and in the HH zone, whereas the LL zone and most 3D variables exhibited multiple directional changes. Spatial-block bootstrap analysis examined 40 full-sample zero-crossing candidates, of which 39 met the predefined stability criteria; these ranges represent model-derived directional transitions rather than ecological thresholds or causal planning standards. The findings demonstrate thermal-context-dependent, model-based associations between 2D and 3D plant morphology and street-scale LST, while emphasizing that model performance and some importance rankings are spatially sensitive and require local validation.

## 1. Introduction

Rapid urbanization has significantly transformed the underlying surfaces of urban areas, as natural land covers are progressively supplanted by buildings, roads, and other impervious materials, thereby exacerbating issues related to the urban thermal environment [1,2,3]. Land surface temperature (LST) is widely used to characterize spatial variation in surface thermal conditions across land-cover and urban-morphology settings [4]. Within the built environment, streets are both transport corridors and frequently used public spaces; one street-tree study noted that streets constitute more than 80% of public space in developed cities [5]. Urban vegetation moderates heat through shading, transpiration, and changes in surface energy exchange, while cooling also depends on green-space configuration and canopy quality [6,7]. A global synthesis of 182 studies across 17 climates and 110 cities or regions found that urban trees could lower pedestrian-level temperatures by up to 12 °C, but their cooling efficacy varied with background climate, urban morphology, and tree traits [8]. Beyond thermal regulation, field data from 28 U.S. cities and six states yielded national estimates of 643 million tonnes of carbon stored and 25.6 million tonnes sequestered annually by urban trees [9]. Therefore, elucidating the relationship between the spatial morphology of street-level vegetation and LST is essential for effective urban thermal management, optimization of street greening, and the advancement of targeted urban renewal strategies.

Previous research on green-space configuration has predominantly examined LST at city, district, block, or grid scales, emphasizing vegetation coverage, green-space proportion, patch composition, and spatial arrangement [6,10,11]. Common 2D landscape metrics include percentage of landscape, patch density, edge density, aggregation index, and shape index. In Fuzhou, analyses at 360, 510, 720, and 960 m grid scales produced summer OLS R^2^ values ranging from 0.424 to 0.551, demonstrating that estimated morphology–LST relationships depend on analytical scale [12]. A recent block-scale study also summarized prior multiscale tests spanning 13 scales from 30 to 600 m and 10 scales from 60 to 600 m, for which 270 and 60 m, respectively, were identified as appropriate analytical scales [13]. In Wuhan, fractional vegetation cover, water proportion, building density, building height, and building volume density were resampled to 500 m urban blocks, revealing pronounced spatial non-stationarity [3]. These broad-scale studies are essential for characterizing citywide thermal structure, but aggregated units may combine roads, buildings, internal green spaces, and other surface types, thereby limiting direct representation of vegetation encountered along continuous street environments [7,14].

At finer scales, street-view imagery, light detection and ranging (LiDAR), unmanned aerial vehicles (UAVs), and micrometeorological observations have enabled more detailed characterization of pedestrian-visible greenery and canopy structure. Street-view studies quantify GVI, tree-canopy visibility, sky openness, and spatial enclosure from the pedestrian perspective [15,16]. In Shenzhen, 90,588 panoramic images sampled at 50 m intervals were combined with six street-view and eight building-form indicators; the RF model explained 79.07% of summer street-level LST variation [17]. A Washington, D.C. analysis compared GVI and NDVI at 15 m individual-tree, 500 m grid, and census-tract scales and found that the top-view index was more scale-dependent than the street-view measure [18]. At crown scale, a mobile-LiDAR and micrometeorological study conducted over 12 summer days linked improved physiological equivalent temperature primarily to reduced solar radiation and identified leaf area index and crown projected area as key structural attributes [5]. A Wuhan street-canyon simulation parameterized leaf area density, crown diameter, tree height, and trunk height and showed that tree form interacts with canyon geometry [14]. A national analysis across 305 Chinese cities further found that the relationship between tree height and LST depends on surrounding building height [19]. Canopy-cooling reviews likewise emphasize canopy quality, density, health, and water-use strategies beyond canopy cover alone [7]. However, these fine-scale studies commonly focus on individual trees, discrete street-view locations, isolated street segments, or localized canyons. A methodological gap therefore remains between broad grid- or block-based analyses and highly localized plant-structure studies: continuous road-network studies that integrate 2D patch metrics with 3D street- and canopy-level indicators while also testing thermal-zone differences, nonlinear model relationships, and spatial robustness remain comparatively limited.

Recent studies combining 2D and 3D urban indicators have primarily used cities, regular grids, blocks, or urban functional zones as analytical units [13,20,21]. Street-view and crown-structure research has instead centered on discrete image locations or localized field and simulation settings [5,14,18]. Against these designs, this study constructs 42,603 street-scale study units along Wuhan’s road network at 50 m intervals, using a 150 m radius to integrate local vegetation and LST information. The seven plant-form variables comprise 2D metrics (A, P_mean, and P_A) and 3D metrics (GVI, NV_mean, CH_mean, and CH_sd), linking top-down green-space configuration with pedestrian-visible greenery, mean 3D green volume, and canopy-height structure. Anselin Local Moran’s I identifies High–High (HH) and Low–Low (LL) zones within the overall study area, and OLS, RF, and SHAP characterize linear associations, nonlinear model contributions, and model-derived directional changes. Residual spatial diagnostics, buffered spatial cross-validation, spatial-block bootstrap, and repeated spatial validation distinguish original point estimates from patterns reproduced under spatial separation and resampling. The study addresses four questions: (1) How do LR and RF performance differ under the original random holdout and spatially separated validation? (2) Which plant-form variables make the largest contributions to the fitted RF models, and how do their nonlinear SHAP relationships and model-derived zero-crossing ranges vary across the overall study area and the HH and LL zones? (3) How do grouped 2D/3D contribution shares and variable-level importance rankings differ among the three analytical groups? (4) Which model-performance and SHAP-based interpretations remain stable under spatial cross-validation, spatial-block bootstrap, and repeated spatial validation?

## 2. Results

### 2.1. Characteristics of Street-Scale Study Units and Spatial Zoning of Land Surface Temperature

**Figure 1:**
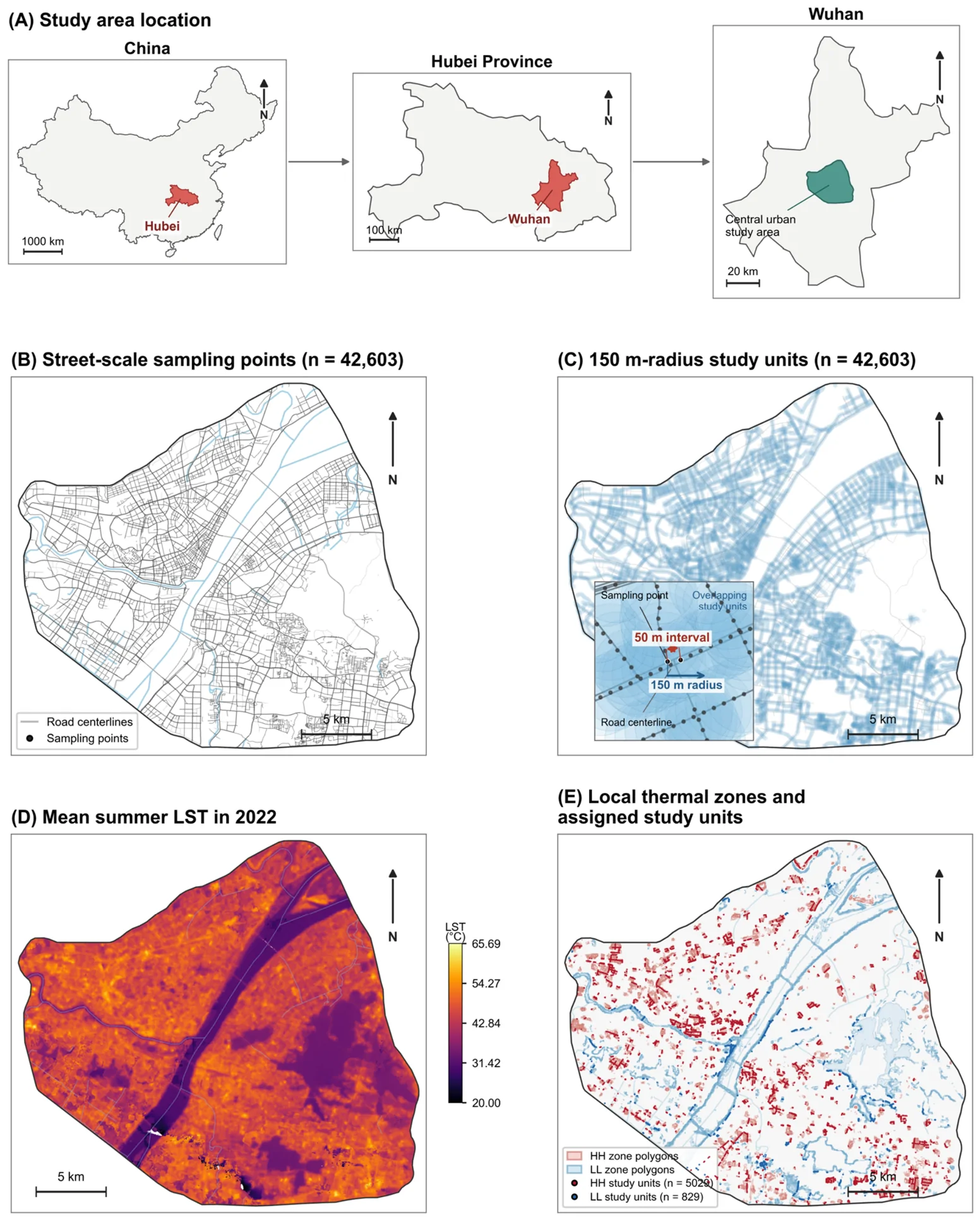
Multiscale location of the study area and spatial distributions of street-scale sampling points, study units, mean summer land surface temperature, and local thermal zones in Wuhan, China. (**A**) Location of Wuhan within China and Hubei Province, and location of the central urban study area within Wuhan; (**B**) street-scale sampling points generated at 50 m intervals along the road network (*n* = 42,603); (**C**) 150 m-radius street-scale study units (*n* = 42,603), with an enlarged inset illustrating the 50 m sampling interval, 150 m study-unit radius, and spatial overlap between adjacent units; (**D**) mean summer land surface temperature during June–August 2022; and (**E**) local thermal zones identified using Anselin Local Moran’s I and the study units assigned to the corresponding zones. HH and LL polygons represent statistically significant High–High and Low–Low thermal zones, respectively, and the overlaid points represent the assigned street-scale study units.

The study area boundary was delineated in accordance with the central urban area defined by the Wuhan Territorial Spatial Master Plan (2021–2035), with the Third Ring Road serving as the principal spatial delimiter (Figure 1A). After cleaning 42,604 candidate records, one invalid record was excluded, leaving 42,603 valid street-scale study units for analysis. Across the overall study area, the mean land surface temperature (LST_mean) was 46.46 °C, with a standard deviation of 2.90 °C, and observed values ranged from a minimum of 23.63 °C to a maximum of 57.55 °C. This 33.93 °C range reflects substantial spatial variation in street-scale LST (Table 1; Figure 1D). Anselin Local Moran’s I identified statistically significant HH and LL zones from the polygonized LST data, and study units were assigned by whether their center points fell within the corresponding polygons (Figure 1E). The HH and LL zones therefore represent local spatial association rather than fixed temperature thresholds. The HH zone comprised 5029 study units, representing 11.80% of the overall sample. Its mean LST_mean was 49.74 ± 1.94 °C, with observed values ranging from 39.70 to 57.55 °C. The LL zone contained 829 study units, accounting for 1.95% of the overall sample, and exhibited a mean LST_mean of 39.50 ± 2.45 °C, with an observed range of 23.63–46.16 °C. The mean LST_mean of the HH zone was 3.29 °C higher than the overall mean, whereas that of the LL zone was 6.96 °C lower. The difference between the mean temperatures of the two zones was 10.25 °C, calculated using the unrounded mean values. Their observed temperature ranges partially overlapped because membership was based on local spatial association with neighboring LST polygons rather than mutually exclusive temperature cutoffs.

**Figure 2:**
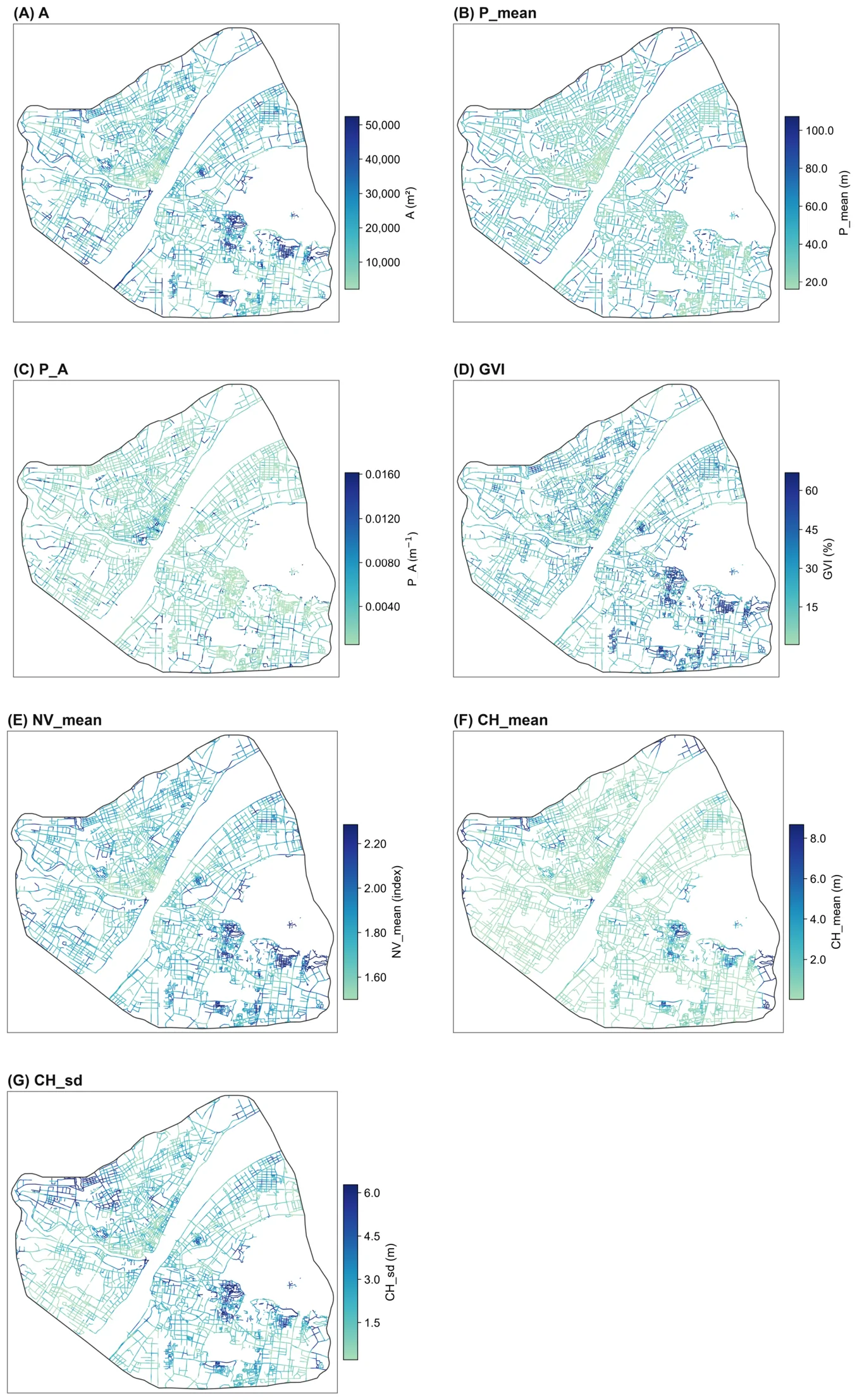
Spatial distributions of plant-form variables across the 42,603 street-scale study units. Panels show (**A**) green-space area (A), (**B**) mean patch perimeter (P_mean), (**C**) perimeter–area ratio of green space (P_A), (**D**) green view index (GVI), (**E**) mean 3D green volume (NV_mean), (**F**) mean canopy height (CH_mean), and (**G**) standard deviation of canopy height (CH_sd). Each panel uses an independent color scale because the variables differ in units and value ranges; within each panel, lighter and darker colors indicate lower and higher values, respectively. For visualization, color limits were defined using the 2nd and 98th percentiles, with values outside this range assigned to the corresponding color-scale endpoints.

Plant-form variables varied substantially across the 42,603 street-scale study units (Figure 2). The LL zone had higher mean values than the HH zone for all seven variables—A, P_mean, P_A, GVI, NV_mean, CH_mean, and CH_sd (Table 1). These descriptive differences encompassed both 2D and 3D plant morphology but did not establish causal thermal effects. Subsequent models were fitted for the overall study area and separately for the HH and LL zones.

### 2.2. Linear Relationships Between Plant-Form Variables and LST

**Figure 3:**
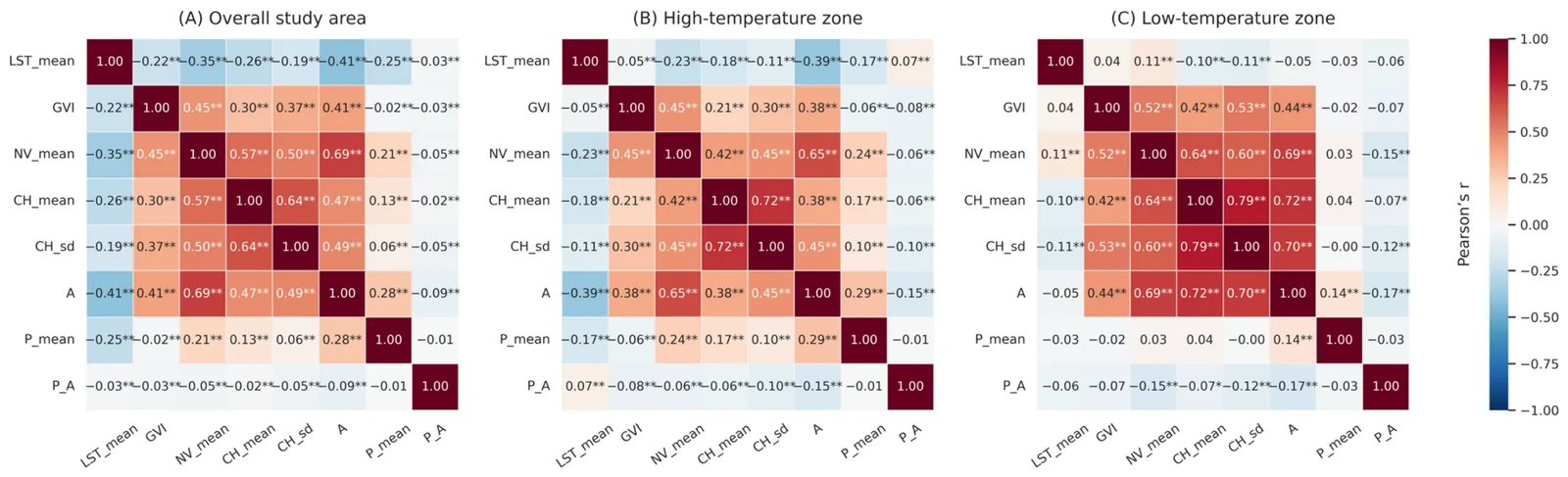
Pearson correlation heatmaps of LST and plant-form variables across the three analytical groups: (**A**) overall study area, (**B**) HH zone, and (**C**) LL zone. The asterisks following the Pearson correlation coefficients indicate statistical significance, with * denoting *p* < 0.05 and ** denoting *p* < 0.01.

Pearson correlation analysis showed predominantly negative bivariate linear associations between LST_mean and the plant-form variables in the overall study area (Figure 3A). A had the strongest negative correlation with LST_mean (r = −0.412), followed by NV_mean (r = −0.350), CH_mean (r = −0.262), P_mean (r = −0.249), GVI (r = −0.224), and CH_sd (r = −0.186); the association for P_A was comparatively weak (r = −0.026). Within the HH zone, A again had the strongest negative correlation with LST_mean (r = −0.386), whereas GVI and P_A had weaker linear associations (Figure 3B). Correlations in the LL zone were generally low, motivating the subsequent multivariable and nonlinear analyses (Figure 3C).

The results of the multiple linear regression analysis indicated that the explanatory capacity of the linear models was limited across all three analytical groups. Specifically, the full-sample ordinary least squares (OLS) models yielded coefficient of determination (R^2^) values of 0.2103 for the overall study area, 0.1763 for the HH zone, and 0.0809 for the LL zone. The variance inflation factor (VIF) values ranged from 1.010 to 2.363 in the overall study area, from 1.031 to 2.327 in the HH zone, and from 1.043 to 3.356 in the LL zone. The maximum VIF values were observed for NV_mean, CH_sd, and CH_mean in the three groups, respectively. Because all VIF values were below 5, severe linear multicollinearity was not detected among the seven plant-form variables. Using conventional OLS inference, all seven coefficients reached the nominal *p* < 0.05 level in the overall study area. Six coefficients were negative, whereas CH_sd had a positive coefficient. In the HH zone, A, CH_mean, and P_mean had nominally significant negative coefficients, whereas GVI and CH_sd had nominally significant positive coefficients; NV_mean and P_A did not reach the nominal significance level. In the LL zone, NV_mean had a nominally significant positive coefficient, whereas CH_mean and CH_sd had nominally significant negative coefficients. Because the residual Global Moran’s I tests identified significant spatial autocorrelation in all three analytical groups, these conventional OLS *p*-values are interpreted as descriptive, model-based quantities rather than spatially robust inferential evidence. Accordingly, the coefficient signs and magnitudes are used to summarize within-sample linear associations.

**Figure 4:**
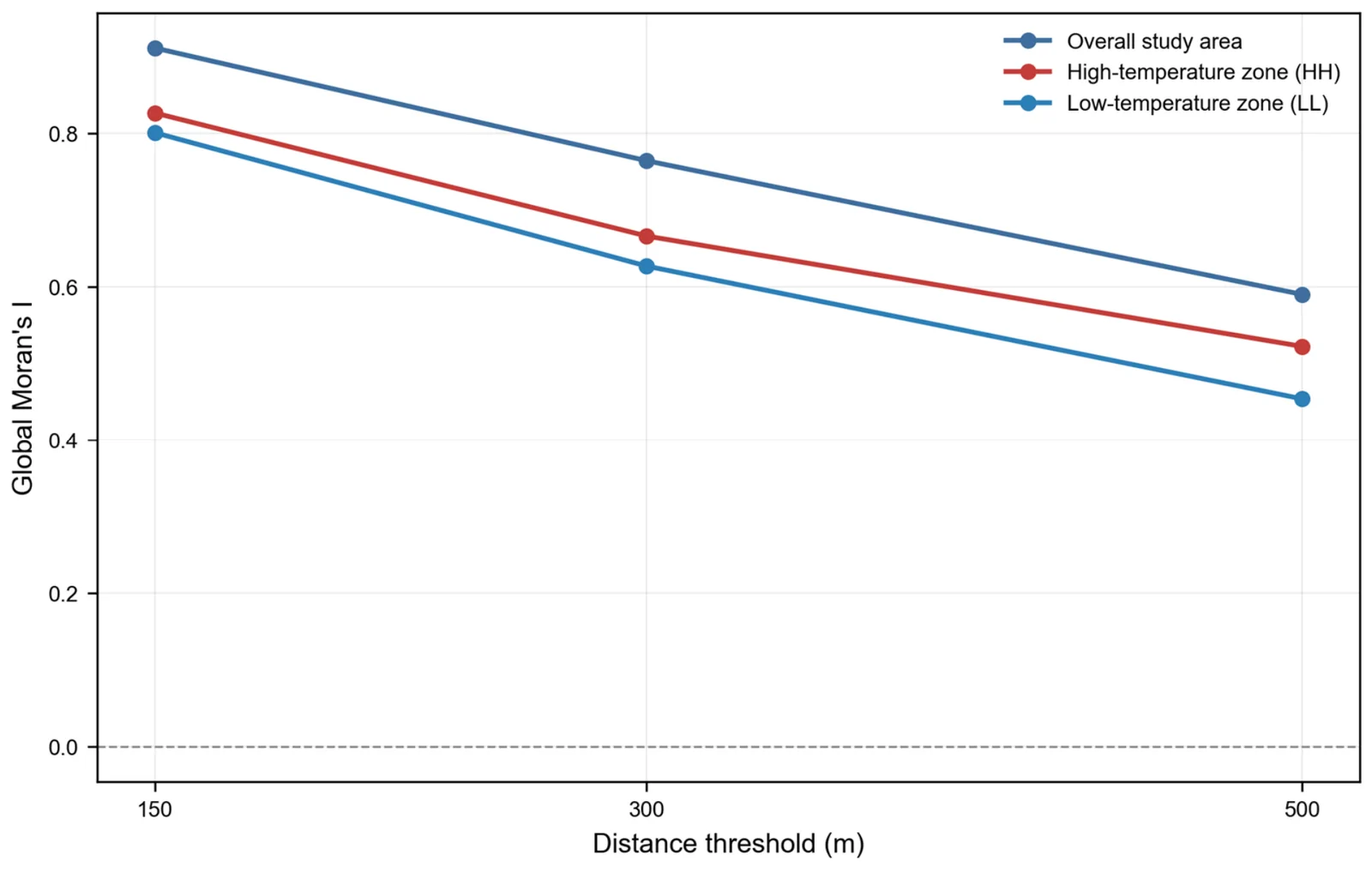
Global Moran’s I of OLS residuals for the overall study area, the HH zone, and the LL zone at distance thresholds of 150, 300, and 500 m. All Moran’s I values were positive and statistically significant at *p* < 0.001. The dashed horizontal line indicates Moran’s I = 0.

Global Moran’s I showed significant positive spatial autocorrelation in the OLS residuals across all three analytical groups and distance thresholds (Figure 4). In the overall study area, Moran’s I decreased from 0.9112 at 150 m to 0.7643 at 300 m and 0.5902 at 500 m. The corresponding values were 0.8265, 0.6663, and 0.5225 for the HH zone, and 0.8009, 0.6271, and 0.4542 for the LL zone. All tests were statistically significant at *p* < 0.001. At distance thresholds of 150, 300, and 500 m, the numbers of isolated study units were 6, 0, and 0 in the overall study area; 79, 42, and 17 in the HH zone; and 48, 32, and 23 in the LL zone, respectively. The decline with distance indicates that residual spatial dependence was strongest among nearby study units but remained evident at broader scales. Because the independence assumption for conventional OLS standard errors was not fully satisfied, the coefficient *p*-values are treated as nominal rather than spatially robust. Buffered spatial cross-validation evaluates predictive robustness under spatial separation but does not correct OLS inferential uncertainty.

### 2.3. Comparative Analysis of Linear Regression and Random Forest Models in Predicting Street-Scale Land Surface Temperature

**Figure 5:**
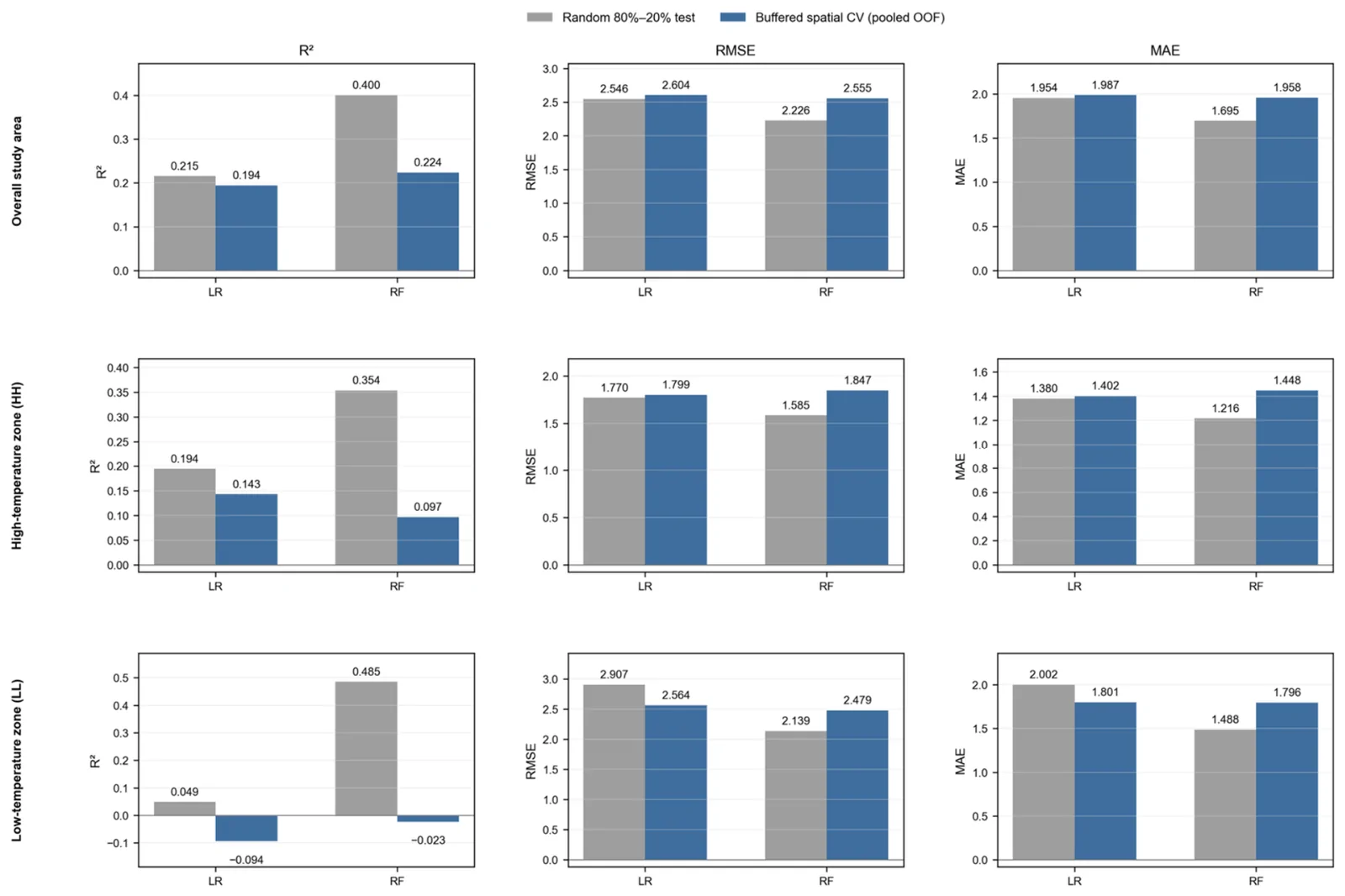
Comparison of model performance under the original random 80–20% train–test split and five-fold buffered spatial block cross-validation. R^2^, RMSE, and MAE are reported for linear regression and random forest models in the overall study area and the HH and LL zones. Spatial cross-validation used 1 km × 1 km blocks and excluded candidate training units located within 300 m of testing units.

LR and RF were fitted with the same explanatory variables and identical training and testing samples. R^2^, RMSE, and MAE were used to compare their predictive performance (Figure 5). Under the original random 80–20% train–test split, RF outperformed LR in the overall study area and the HH and LL zones; five-fold buffered spatial cross-validation produced more conservative and heterogeneous results.

Specifically, within the overall study area, the testing R^2^ value for the linear regression model was 0.2153, whereas the random forest model achieved a substantially higher R^2^ of 0.4002. Correspondingly, RMSE decreased from 2.5458 to 2.2258, and MAE was reduced from 1.9538 to 1.6952. In the HH zone, the random forest model attained an R^2^ of 0.3539, surpassing the linear regression model’s R^2^ of 0.1940; RMSE and MAE similarly declined from 1.7697 and 1.3796 to 1.5845 and 1.2164, respectively. The most notable enhancement was observed in the LL zone, where the R^2^ increased markedly from 0.0488 to 0.4853, accompanied by reductions in RMSE and MAE from 2.9074 and 2.0022 to 2.1387 and 1.4881, respectively. These results indicate that the random forest model captured additional nonlinear variation within the randomly partitioned Wuhan dataset, particularly in the LL zone.

As shown in Figure 5, five-fold buffered spatial cross-validation (spatial CV) produced more conservative performance estimates than the original random train–test split. The spatial CV results were evaluated using pooled out-of-fold (OOF) predictions. In the overall study area, the pooled OOF R^2^ values were 0.1940 for LR and 0.2237 for RF, with corresponding RMSE values of 2.6038 and 2.5554 and MAE values of 1.9872 and 1.9583, respectively. Thus, RF retained only a modest advantage over LR under spatially separated validation. In the HH zone, LR yielded R^2^ = 0.1427, RMSE = 1.7994, and MAE = 1.4024, whereas RF yielded R^2^ = 0.0965, RMSE = 1.8473, and MAE = 1.4478. In the LL zone, LR yielded R^2^ = −0.0942, RMSE = 2.5635, and MAE = 1.8007, while RF yielded R^2^ = −0.0231, RMSE = 2.4788, and MAE = 1.7964. The negative pooled OOF R^2^ values indicate performance below the constant-mean benchmark calculated from the pooled OOF observations. Overall, the RF advantage under random partitioning was not maintained consistently after spatial separation, indicating sensitivity to the spatial configuration of validation samples rather than model failure.

### 2.4. Differences and Spatial Stability of Plant-Form Variable Importance Across Analytical Groups

**Figure 6:**
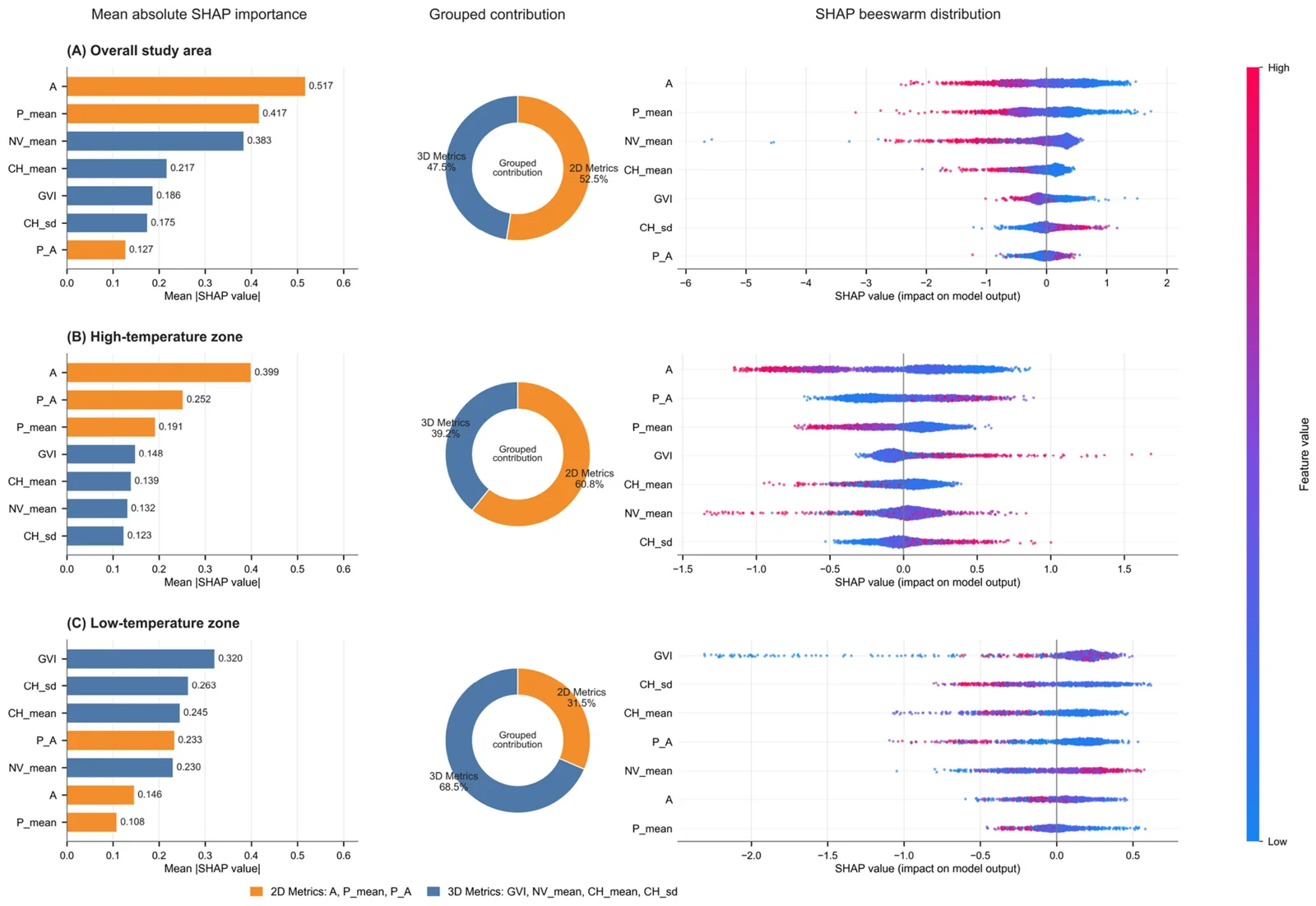
SHAP-based variable importance, grouped contribution shares, and feature effects of plant-form variables across the three analytical groups. Rows represent (**A**) the overall study area, (**B**) the HH zone, and (**C**) the LL zone. In each row, the left panel shows the mean absolute SHAP importance of each plant-form variable, the middle panel shows the grouped point-estimate contribution shares of 2D and 3D metrics derived from the original RF models, and the right panel shows the SHAP beeswarm distribution.

SHAP analysis of the original fitted RF models revealed distinct contribution patterns across the three analytical groups. Within the overall study area, A, P_mean, and NV_mean had the largest mean absolute SHAP values, at 0.517, 0.417, and 0.383, respectively (Figure 6A). These variables therefore made the largest average contributions to the fitted overall RF model. In the original overall RF model, 2D metrics accounted for 52.5% of the aggregated mean absolute SHAP contribution, compared with 47.5% for 3D metrics. This near-balanced point estimate was evaluated further through repeated buffered spatial validation rather than interpreted as stable 2D predominance. The beeswarm plot shows that the SHAP values of A, P_mean, and NV_mean occurred on both sides of zero, indicating that their contributions to RF-predicted LST_mean changed in direction across the observed value ranges and spatial contexts.

In the original fitted HH RF model, A, P_A, and P_mean had the three largest mean absolute SHAP values, at 0.399, 0.252, and 0.191, respectively (Figure 6B). The grouped 2D contribution share was 60.8%, compared with 39.2% for the 3D metrics. These model-based results indicate that RF-predicted LST_mean in the HH zone was more strongly associated with green-space area, perimeter–area ratio, and mean patch perimeter than with the 3D variables considered. The beeswarm distributions further show that the contributions of these variables changed sign across their observed values, rather than representing uniformly warming or cooling effects.

Conversely, the LL zone exhibited a distinct contribution profile. In the original fitted LL RF model, GVI, CH_sd, and CH_mean had the three largest mean absolute SHAP values, at 0.320, 0.263, and 0.245, respectively (Figure 6C). The grouped contribution of 3D metrics reached 68.5%, compared with 31.5% for 2D metrics. These point estimates indicate that vertical vegetation structure and pedestrian-visible greenery accounted for a larger share of the fitted LL model than green-space area and patch morphology. However, these variable-level rankings describe the original fitted model and should not be interpreted as a stable ordering across alternative spatial samples; the repeated spatial validation results are reported separately below.

**Figure 7:**
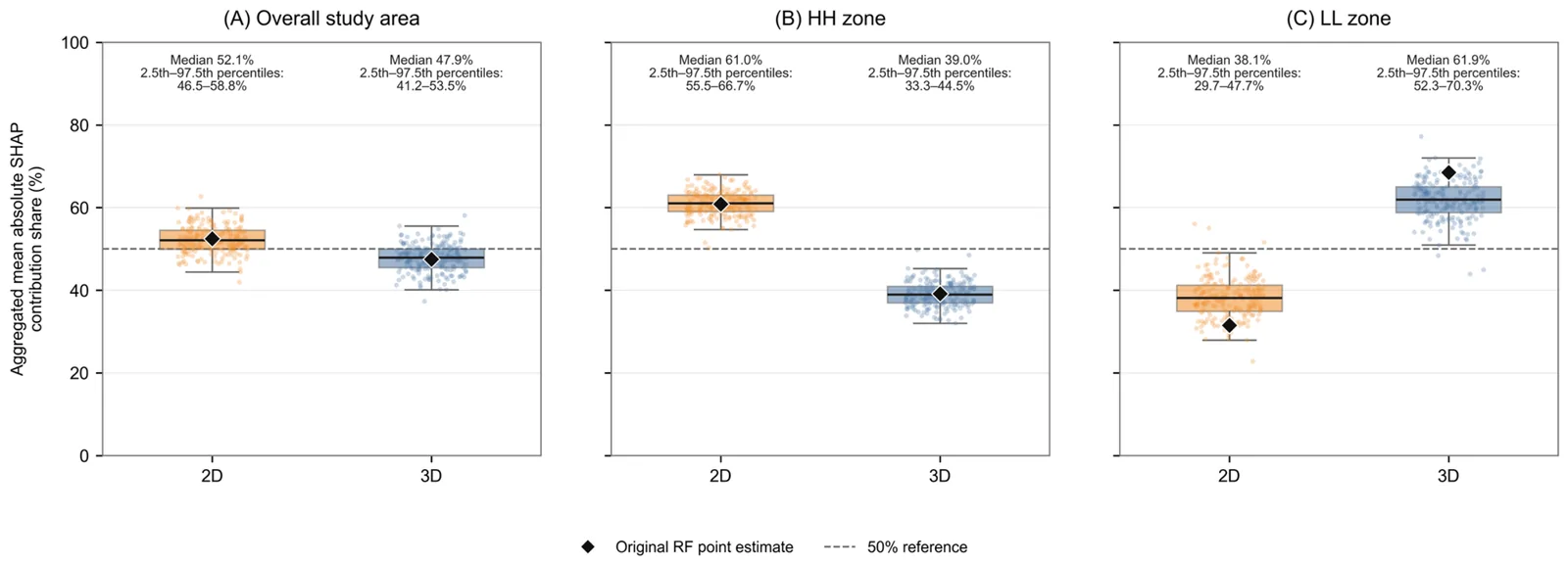
Stability of aggregated mean absolute SHAP contribution shares under repeated buffered spatial validation. Panels represent (**A**) the overall study area, (**B**) the HH zone, and (**C**) the LL zone. Orange and blue distributions represent the grouped contribution shares of 2D variables (A, P_mean, and P_A) and 3D variables (GVI, NV_mean, CH_mean, and CH_sd), respectively, across 200 valid spatial repetitions. RF models were refitted in each repetition, and exact TreeSHAP values were calculated for the spatially separated test samples. Black diamonds indicate the original RF point estimates, and the dashed horizontal line indicates the 50% reference. The reported ranges are empirical 2.5th–97.5th percentiles rather than causal confidence intervals.

Repeated buffered spatial validation further showed that the relative contribution structure of 2D and 3D variables differed in stability among the three analytical groups (Table 2; Figure 7). In the overall study area, the median aggregated mean absolute SHAP contribution share of 2D variables was 52.1%, with a 2.5th–97.5th percentile range of 46.5–58.8%. Because this range crossed the 50% reference, the slight 2D predominance observed in the original RF point estimate was sensitive to spatial resampling. In the HH zone, the 2D contribution share had a median of 61.0% and a percentile range of 55.5–66.7%, indicating stable 2D relative predominance. Conversely, in the LL zone, the 3D contribution share had a median of 61.9% and a percentile range of 52.3–70.3%, supporting stable 3D relative predominance.

**Figure 8:**
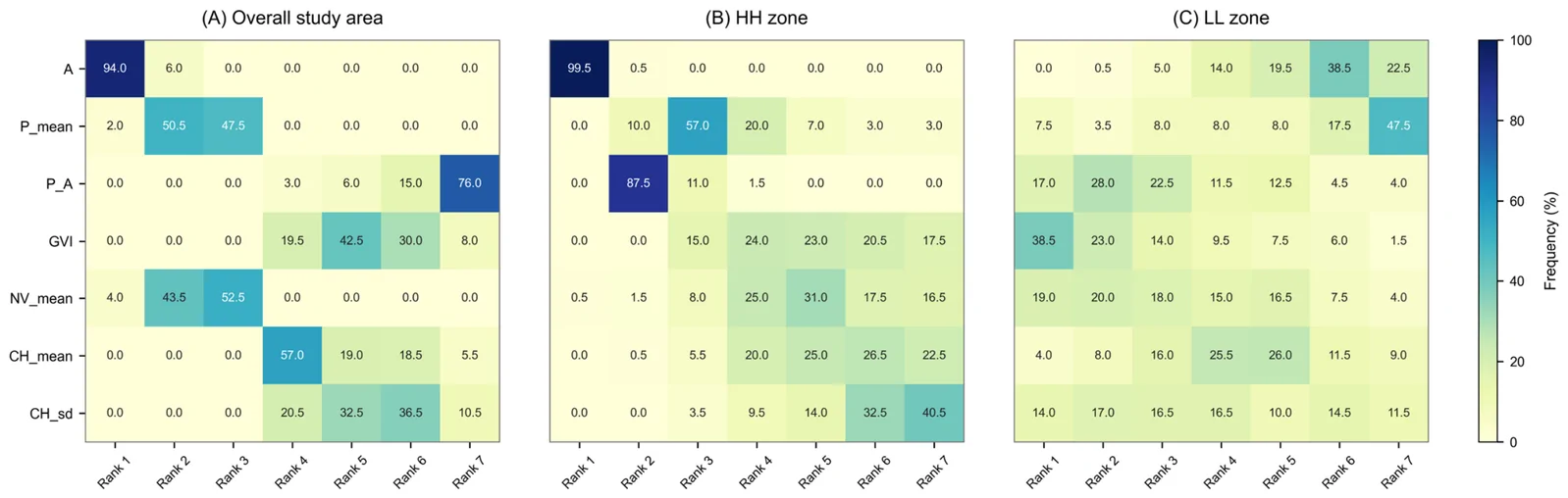
Rank-frequency distributions of plant-form variables under repeated buffered spatial validation. Panels represent (**A**) the overall study area, (**B**) the HH zone, and (**C**) the LL zone. Each cell reports the percentage of the 200 valid repetitions in which a variable occupied the corresponding mean absolute SHAP importance rank. Rank 1 represents the largest mean absolute SHAP contribution. Darker cells indicate higher ranking frequencies. The results characterize the stability of relative variable importance across spatial resamples and do not represent statistical significance or causal effects.

Variable-level rank stability also differed markedly among the analytical groups (Figure 8). In the overall study area, A ranked first in 94.0% of the repeated models, while P_mean and NV_mean were consistently ranked within the top three. In the HH zone, A ranked first in 99.5% of the repetitions, and P_A was placed within the top three in 98.5% of the repetitions, indicating comparatively stable variable-level importance. In contrast, the LL zone exhibited substantially greater rank variability. GVI ranked first in 38.5% of the repetitions, followed by NV_mean, P_A, and CH_sd at 19.0%, 17.0%, and 14.0%, respectively. The mean Spearman correlation between repeated and original variable rankings was only 0.410 in the LL zone. Thus, although the grouped contribution of 3D variables was relatively stable in the LL zone, the predominance of any individual variable was not.

### 2.5. Nonlinear SHAP Dependence Relationships and Zero-Crossing Ranges

**Figure 9:**
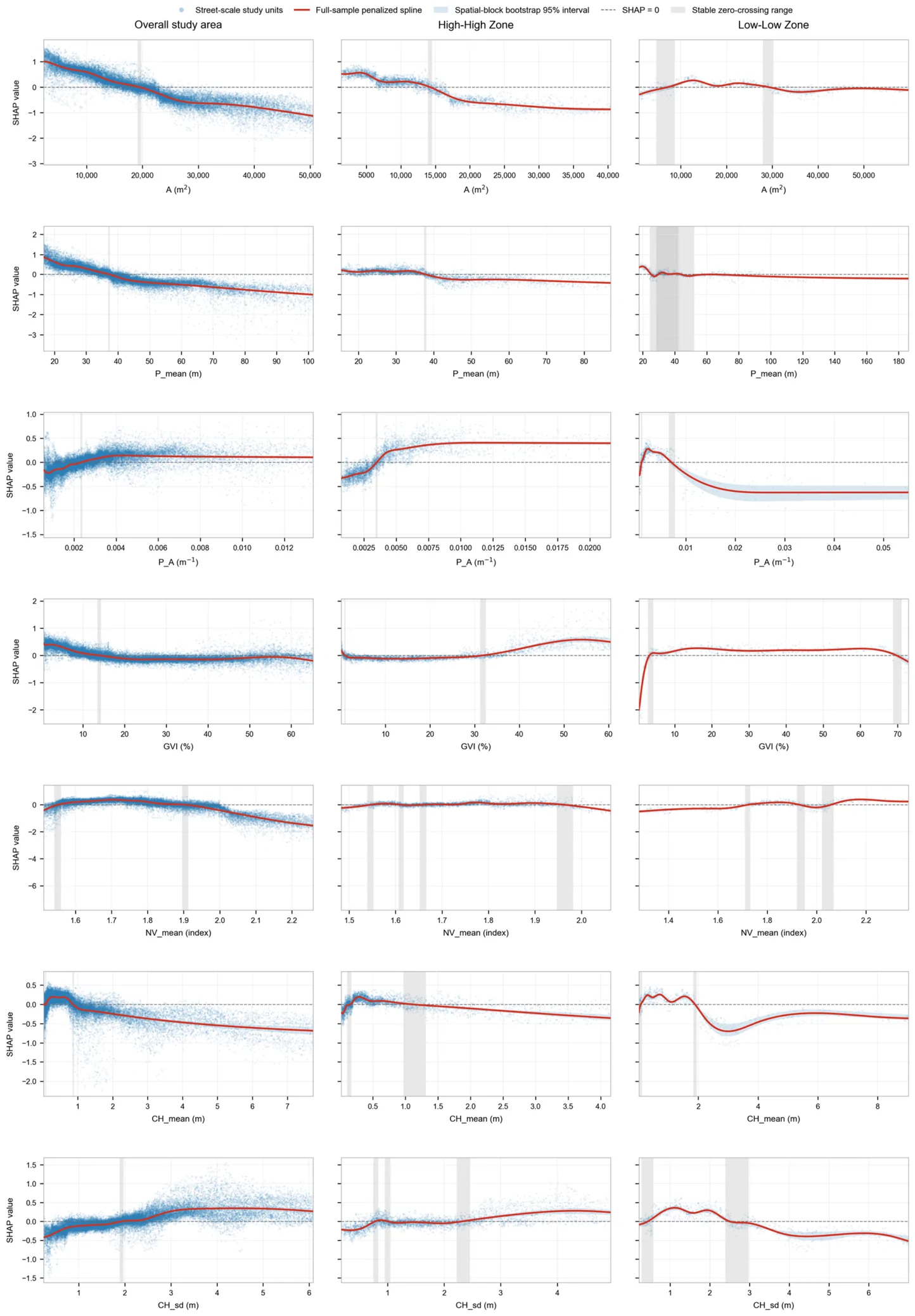
SHAP dependence relationships and spatial-block bootstrap uncertainty for plant-form variables across the three analytical groups. Columns represent the overall study area, the HH zone, and the LL zone, while rows represent A, P_mean, P_A, GVI, NV_mean, CH_mean, and CH_sd. Light-blue points represent the SHAP dependence data for individual street-scale study units. Red curves represent penalized cubic B-spline trends fitted to the SHAP dependence data for all valid study units, and blue bands represent the pointwise 95% intervals obtained from 500 spatial-block bootstrap resamples using 1 km × 1 km blocks. Horizontal dashed lines denote SHAP = 0, and gray vertical bands indicate stable model-derived zero-crossing ranges. Positive and negative SHAP values indicate contributions that increase and decrease the RF model output relative to its baseline prediction, respectively, and should not be interpreted as causal effects.

The nonlinear relationships between the plant-form variables and LST_mean were further examined using SHAP dependence data, penalized cubic B-spline smoothing, and 500 spatial-block bootstrap resamples. Among the 40 full-sample zero-crossing candidates identified across the three analytical groups, 39 satisfied the predefined stability criteria. The corresponding bootstrap medians and 95% intervals are reported in Table 3 and visualized as gray vertical bands in Figure 9. These ranges indicate changes in the direction of the variables’ contributions to the RF model output rather than causal or universally transferable ecological thresholds.

For the 2D planar metrics, A and P_mean showed relatively clear positive-to-negative transitions in both the overall study area and the HH zone. In the overall study area, the bootstrap median zero-crossing values of A and P_mean were 19,455 m^2^ and 37.22 m, with 95% intervals of 19,147–19,786 m^2^ and 37.00–37.42 m, respectively. The corresponding values in the HH zone were 14,213 m^2^ for A and 37.75 m for P_mean. P_A exhibited negative-to-positive transitions at 0.002342 m^−1^ in the overall study area and 0.003494 m^−1^ in the HH zone. In the LL zone, A displayed two stable directional transitions, centered at 7161 and 29,194 m^2^. The three P_mean candidates in this zone had overlapping intervals spanning approximately 24.35–52.20 m, indicating weak multistage fluctuations rather than clearly separable transition points. P_A also showed two directional changes in the LL zone, centered at approximately 0.000998 and 0.007098 m^−1^.

The 3D variables generally exhibited more complex and zone-dependent response patterns. In the overall study area, GVI changed from positive to negative SHAP contributions at a bootstrap median of 13.94%, whereas NV_mean showed negative-to-positive and positive-to-negative transitions at 1.551 and 1.905, respectively. CH_mean had stable directional transitions at 0.085 and 0.869 m; an additional low-value candidate at approximately 0.033 m did not satisfy the stability criteria and was therefore not retained. CH_sd changed from negative to positive contributions at approximately 1.923 m.

In the HH zone, GVI showed two transitions, at approximately 0.934% and 31.92%, while NV_mean exhibited four successive directional changes between approximately 1.546 and 1.964. CH_mean changed direction at approximately 0.155 and 1.108 m, and CH_sd exhibited three transitions centered at 0.776, 0.998, and 2.322 m. In the LL zone, GVI showed stable transitions at 3.21% and 69.75%. NV_mean exhibited three directional changes at approximately 1.720, 1.931, and 2.056, while CH_mean changed direction at 0.075 and 1.891 m. CH_sd displayed two transitions centered at approximately 0.434 and 2.498 m.

Overall, the SHAP response curves demonstrated that the associations between plant-form variables and RF-predicted LST_mean were neither uniformly linear nor consistently monotonic. A and P_mean exhibited comparatively distinct positive-to-negative transitions in the overall study area and the HH zone, whereas most 3D variables and the LL-zone responses contained multiple directional changes. The relatively narrow bootstrap intervals of many transitions indicate that their locations were reproducible under the adopted spatial resampling procedure. Nevertheless, these ranges remain conditional on the fitted RF models, the 2022 Wuhan dataset, and the current definition of street-scale study units.

## 3. Discussion

### 3.1. Methodological Advancement: Integrating Street-Scale 2D and 3D Plant Morphology with Thermal Environment Variability

The first methodological contribution is a continuous street-scale framework positioned between city- or block-scale grids and highly localized individual-tree or street-canyon studies. Sampling points at 50 m intervals and 150 m-radius study units follow the road network, allowing 2D green-space metrics (A, P_mean, and P_A) and 3D plant-form metrics (GVI, NV_mean, CH_mean, and CH_sd) to share the same spatial support. This design links top-down patch morphology with pedestrian-visible greenery, mean 3D green volume, and canopy-height structure while retaining continuity for citywide comparison. Recent city-, block-, and functional-zone studies have combined 2D and 3D indicators at broader spatial supports [11,21]. Street-view and individual-tree research has provided detailed pedestrian- and crown-level evidence at discrete or localized units [5,18]. The present framework therefore provides an intermediate-scale representation aligned with street greening and pedestrian street environments.

The second methodological contribution is an integrated workflow for HH/LL zoning, model comparison, spatial diagnostics, and SHAP stability assessment. Anselin Local Moran’s I defines the HH and LL zones without fixed temperature cutoffs; OLS and RF distinguish average linear associations from nonlinear predictive relationships; and residual Global Moran’s I and buffered spatial cross-validation separate within-area fit from spatial transferability. SHAP analysis combines original importance and dependence estimates with grouped 2D/3D contribution stability, variable-rank stability, and zero-crossing uncertainty under repeated spatial validation and spatial-block bootstrap. The workflow therefore evaluates both what the fitted models capture and which interpretations remain reproducible under spatial resampling, without treating model outputs as causal or universally stable.

### 3.2. Significance of Nonlinear Models and Street-Scale Study Units for Explaining LST

From the standpoint of model selection, the findings indicate that street-scale plant morphology cannot be fully represented by a single linear response framework. Prior research offers more detailed evidence supporting this limitation. For instance, Chen et al. demonstrated that horizontal and vertical tree-canopy structures differentially explained daytime and nighttime LST, suggesting that the association of identical vegetation-structure variables may vary across thermal processes rather than remaining constant under a single linear coefficient [22]. From a modeling perspective, Liu et al. further highlighted that conventional correlation and regression methods frequently fail to account for the nonlinear and interactive dynamics of LST, whereas ensemble learning models improve response characterization and SHAP supports model interpretation [23]. Similarly, Yuan et al. underscored the complexity and multidimensionality of the relationship between 2D and 3D urban morphology and LST; although machine-learning approaches have begun to examine range-dependent responses, interactions among 2D and 3D variables remain inadequately investigated, with SHAP offering a useful means of interpreting variable contributions and interactions [24]. Accordingly, the present study employed RF not to infer physical causation, but to test whether the fitted vegetation–LST relationships contained nonlinear, range-dependent, and directionally variable model contributions beyond average linear associations.

Linear models often inadequately represent spatial non-stationarity and complex LST relationships [16,25]. Here, RF and SHAP were used to characterize nonlinear, range-dependent model relationships within a predictor set restricted to street-scale 2D and 3D plant morphology [20,26]. A SHAP value quantifies how a variable shifts an RF prediction relative to the model baseline; it does not establish physical causation. Likewise, model-derived zero-crossing ranges describe sign changes in fitted SHAP contributions rather than ecological thresholds, universal design standards, or minimum greening requirements. Spatial CV further limits interpretation to within-area model patterns unless independent spatial transferability is demonstrated.

The analytical unit also shapes LST interpretation. Regular grid or block units facilitate citywide comparisons of urban morphology, green-space patterns, and thermal environments [13,20], but they may combine roads, buildings, internal green spaces, and other surfaces, obscuring street-level vegetation. Road-based sampling and street-view imagery instead represent pedestrian-visible greenery and the conditions encountered during daily mobility [27]. Street-canyon studies further show that LAD, crown diameter, tree height, and under-branch height interact with canyon geometry [14]. Street-scale study units therefore complement, rather than replace, grid-based analyses by aligning plant-form metrics with road-space context.

These comparisons also make clear that analytical scale is not neutral. In Fuzhou, 360, 510, 720, and 960 m grids yielded scale-dependent vegetation–LST relationships, with the strongest reported model fit at 510 m [12]; a Washington, D.C., study compared a 15 m individual-tree buffer, a 500 m grid, and census tracts and found that greenery–temperature associations changed with spatial support [18]. Other urban thermal studies have used block-scale units [13] and 150 m grids [23]. Against this range, the present 150 m radius should be understood as an intermediate, study-specific spatial support rather than a universally optimal scale. It preserves sensitivity to the plant environment near streets while encompassing multiple 30 m Landsat pixels and allowing A, P_mean, P_A, GVI, NV_mean, CH_mean, and CH_sd to be summarized on a common support; the 50 m sampling step then moves this support continuously along the road network. Alternative buffer radii could produce different estimated relationships and remain a priority for sensitivity analysis.

The contrast between random and spatial validation highlights an important distinction between within-area prediction and spatial transferability. Under random partitioning, nearby study units with similar vegetation and thermal characteristics may occur in both the training and testing datasets, whereas buffered spatial CV evaluates predictions for locations separated from the training samples. Under spatial CV, RF retained only a modest advantage over LR in the overall study area, performed below LR in the HH zone, and produced a negative pooled out-of-fold (OOF) R^2^ together with LR in the LL zone. Because the random holdout and spatial CV used different resampling structures and testing samples, these differences should not be attributed solely to spatial leakage. Rather, they show that the nonlinear relationships captured by RF were more reliable for within-area prediction than for spatial extrapolation. In particular, a negative pooled OOF R^2^ indicates that the corresponding spatial predictions performed worse than the constant-mean baseline calculated for the pooled OOF observations. The relatively modest R^2^ values should also be interpreted in relation to the deliberately restricted predictor set: the models included only plant-form variables, whereas street-scale LST is additionally shaped by building morphology, road geometry, proximity to water bodies, surface materials, traffic conditions, and meteorological factors. The models were therefore designed to compare linear and nonlinear representations of vegetation–LST relationships, not to provide an exhaustive prediction of urban thermal conditions. Their transferability is consequently limited to the sampled Wuhan context and requires independent validation before application to other locations, years, or cities.

More specifically, omitted built-form processes may help explain both the modest model fit and the remaining spatial structure. Building density, building height, and sky openness can modify solar exposure, radiative exchange, shading, ventilation, and heat storage [17], while the thermal contribution associated with tree height can vary with surrounding building height [19]. Road geometry, surface properties, and hydrological setting may add further spatially structured variation. Because these processes were not measured systematically here, they may partly account for unexplained LST variation but cannot be identified as the cause of the observed residual Moran’s I.

The LR–RF comparison was intentionally focused: LR provides a transparent linear benchmark, whereas RF accommodates nonlinearities and interactions among heterogeneous predictors and can be interpreted consistently with TreeSHAP [28,29]. Prior multi-model evidence does not indicate a universal algorithm ranking. At the block scale, BRT outperformed RF for diurnal LST attribution [13], whereas another urban thermal comparison reported that RF achieved better test-set performance than XGBoost and LightGBM but was outperformed by TabPFN [26]. This mixed evidence supports retaining RF as a competitive and interpretable nonlinear learner for the present questions, not as a claim that it is universally superior.

### 3.3. Differentiated Roles of 2D Planar Morphology and 3D Vertical Structure

SHAP analysis identified zone-dependent differences between 2D and 3D plant morphology. In the original overall RF model, 2D variables accounted for 52.5% of the aggregated mean absolute SHAP contribution and 3D variables for 47.5%. Repeated buffered spatial validation yielded a median 2D share of 52.1% with a 2.5th–97.5th percentile range of 46.5–58.8%; because this range crossed 50%, the overall study area is better characterized as near-balanced than as having stable 2D predominance. By contrast, the HH-zone 2D share and LL-zone 3D share remained above 50% across their percentile ranges (Table 2; Figure 7). Citywide and seasonal studies associate green-space area, patch configuration, and aggregation with LST across different urban and landscape contexts [6,10,12]. Multiscale and block-based analyses further show that these associations vary with analytical scale and local spatial setting [13,20,30]. In the original HH RF model, 2D and 3D metrics accounted for 60.8% and 39.2%, respectively (Figure 6), indicating prominence within the fitted model rather than a causal fragmentation or connectivity mechanism, neither of which was measured directly.

In the original HH RF model, A, P_A, and P_mean had the largest mean absolute SHAP values (0.3991, 0.2518, and 0.1915, respectively; Figure 6). A quantifies green-space area, P_mean the mean patch perimeter, and P_A perimeter relative to area. In the less-green HH context, these metrics may represent contrasts in vegetation amount and configuration more directly than the available 3D metrics. Their rankings identify important model contributors, not causal LST drivers. Reported associations of green-space size, edge attributes, and spatial arrangement with LST in densely built environments provide contextual support [6,10,12]. A separate SHAP-based study likewise reported nonlinear contributions and interactions among 2D and 3D urban-form variables [24].

The model-derived zero-crossing ranges further describe fitted nonlinear relationships. For A, the stable positive-to-negative ranges were 19,147–19,786 m^2^ in the overall study area and 13,941–14,507 m^2^ in the HH zone; the corresponding P_mean ranges were 37.00–37.42 m and 37.45–38.08 m (Figure 9; Table 3). These transitions are changes in contribution direction for RF-predicted LST_mean, not minimum greening requirements or causal cooling thresholds. A study of 14 Shanghai pocket parks linked canopy coverage with lower LST, air temperature, and Universal Thermal Climate Index (UTCI) [31], providing contextual evidence but not validation of the present ranges because the analytical units, outcomes, and models differ.

The LL zone showed greater nonlinearity and context dependence among 3D metrics. In the original LL RF model, 3D and 2D metrics accounted for 68.5% and 31.5% of the aggregated mean absolute SHAP contribution, respectively (Figure 6), and repeated validation retained a median 3D share above 50% (Table 2; Figure 7). This grouped prominence does not establish a sequential shift from green-space quantity to vegetation quality. Studies of pocket parks, roadside trees, and individual crowns have related vertical canopy structure, tree height, LAI, canopy volume, and crown morphology to thermal conditions and comfort [31,32,33]. Reviews likewise emphasize canopy quality, density, leaf-area characteristics, and water-use strategy rather than canopy cover alone [7]. These mechanisms provide context for interpretation but were not directly tested by the fitted SHAP models.

The 3D indicators used here should nevertheless be interpreted as partial proxies for vegetation structure rather than complete descriptors of tree functional traits. Urban-tree cooling depends not only on tree size and canopy geometry but also on species-specific foliage and physiological characteristics. Differences between deciduous and evergreen trees, as well as variation in leaf thickness, texture, leaf area, canopy density, stomatal behavior, and water-use strategy, can modify the balance between solar-radiation interception, shading, and transpiration [8]. Denser or larger crowns can increase the area and duration of shade, whereas differences in leaf-area characteristics and canopy density affect the penetration of solar radiation through the canopy and the amount of foliage available for evapotranspiration. Species-specific physiological traits, particularly stomatal regulation and water-use strategy, may further alter transpiration rates under hot and humid conditions. Consequently, trees with comparable height or visible canopy extent may still produce different thermal effects because their foliage structure and physiological functioning differ. Field measurements in a subtropical city likewise showed that crown-scale characteristics were closely associated with under-canopy thermal conditions: 10% increases in crown volume index and total canopy index were associated with 0.67 and 0.83 °C lower mean radiant temperature, respectively, after other predictors were held constant [34]. Therefore, the relatively high grouped contributions of GVI, CH_mean, and CH_sd in the LL zone should not be interpreted as evidence that canopy height or vertical complexity alone determines cooling. Species composition, leaf traits, crown characteristics, and physiological properties may partly account for thermal variation that is not represented by the structural variables used in this study.

In the original LL RF model, GVI, CH_sd, and CH_mean contributed more than A and P_mean (Figure 6), and the LL zone had higher means for all seven plant-form variables than the HH zone (Table 1). One possible interpretation is that the fitted model assigned greater relative importance to visible greenery and canopy structure within this greener context; the analysis does not test whether baseline green-space quantity caused that redistribution. GVI represents pedestrian-visible greenery, whereas CH_mean and CH_sd describe mean canopy height and its variability. This distinction is consistent with evidence that GVI and NDVI capture pedestrian-view and top-down vegetation dimensions, respectively [18]. Variable-level importance was nevertheless unstable: the mean Spearman correlation between repeated and original ranks was 0.410, and no variable consistently ranked first. Thus, stable grouped 3D predominance in the LL zone coexisted with redistribution of SHAP importance among individual predictors.

The HH and LL zones therefore show different model contribution structures, not a universal variable hierarchy or sequential cooling mechanism. Planar green-space amount and configuration were more prominent in the fitted HH model, whereas visible greenery and canopy structure were more prominent in the fitted LL model. Neither pattern implies that increasing a single metric will necessarily reduce LST. A national study of 305 Chinese cities found a segmented tree-height–LST relationship at comparable canopy cover and dependence on surrounding building height [19], consistent with interpreting CH_mean and CH_sd as context-dependent model contributors rather than variables governed by universal cutoffs.

The cross-zone results themselves demonstrate that these directional transitions are context-dependent. The fitted A transition shifted between the overall study area and the HH zone and split into two stable transitions in the LL zone; P_mean was comparatively distinct in the overall and HH models but formed overlapping multistage ranges in the LL model, while GVI and the canopy variables also exhibited zone-specific response structures (Figure 9; Table 3). Beyond this within-city evidence, background climate, evapotranspirative demand, urban density, canyon geometry, sky openness, and ventilation can alter thermal responses [7,17,19]. Vegetation species and functional traits, canopy configuration, observation season, and analytical scale can further modify radiation, airflow, and water-use pathways [8,18,35]. Accordingly, the Wuhan zero-crossing ranges should be interpreted as context-dependent, model-derived directional transitions whose locations may shift under different climatic, morphological, vegetation, temporal, or analytical conditions, rather than as portable thresholds, ecological constants, or planning standards.

The overall fitted RF also illustrates a model-based complementarity between horizontal and vertical plant morphology. A and P_mean changed from positive to negative SHAP contributions beyond their respective transition ranges, and GVI changed from positive to negative at approximately 13.94%, whereas CH_sd changed from negative to positive at approximately 1.923 m. Thus, lower RF-predicted LST corresponded, within different portions of the fitted relationships, to greater horizontal green-space amount or configuration and visible greenery, together with lower-to-moderate canopy-height variability rather than unrestricted increases in every metric. Horizontal metrics represent vegetated amount, patch configuration, shading opportunity, and vegetation–impervious interfaces [20,24]. GVI, NV_mean, CH_mean, and CH_sd characterize pedestrian-visible greenery, green volume, vertical radiation interception, shade distribution, and airflow-related structure [22,31,35]. Their pathways are therefore partly distinct and may complement one another, but the fitted associations do not imply that any prescribed combination will guarantee cooling or that the observed transitions are causal.

The non-monotonic CH_sd response can be interpreted cautiously in light of studies of vertical canopy structure rather than as direct validation of CH_sd itself. Research using foliage height diversity and vertical foliage distribution has reported nonlinear cooling responses and indicates that canopy layering may alter both shade penetration and airflow; pocket-park evidence likewise suggests that evenly distributed multilayer foliage can impede ventilation and produce overlapping foliage that weakens thermal benefits [31,35]. Because foliage height diversity and vertical canopy configuration are not equivalent to the canopy-height standard deviation used here, these competing shading and ventilation processes may only partly explain the observed SHAP pattern. The present study did not measure radiation, airflow, or transpiration directly; therefore, this interpretation should be regarded as a plausible ecological and aerodynamic explanation rather than a confirmed causal mechanism.

### 3.4. Planning Implications for Vegetation Forms

For planning, the findings support zone-specific, locally validated assessment rather than a uniform greening prescription. The HH zone had lower means for all seven plant-form variables than the LL zone, and repeated validation supported stable relative predominance of the 2D group; the LL zone showed stable relative predominance of the 3D group. These patterns identify candidate dimensions for local assessment, not causal design rules. Because fragmentation, connectivity, and intervention effects were not measured directly, the results cannot diagnose a single spatial mechanism or prescribe an exact design.

For the HH zone, 2D green-space morphology is a candidate priority for location-specific evaluation. A, P_A, and P_mean had comparatively large mean absolute SHAP contributions in the original HH RF model, and A and P_mean had stable positive-to-negative zero-crossing ranges (Figure 9; Table 3). Field evaluation could therefore examine effective green-space area, patch configuration, and vegetation–impervious-surface interfaces through measures such as connected roadside green belts, residual roadside or setback spaces, and refined patch boundaries. These are testable planning directions, not causal thresholds or guaranteed site-level cooling outcomes.

For the LL zone, maintaining 3D vegetation structure is a candidate management direction. The original LL RF model assigned comparatively large contributions to GVI, CH_sd, and CH_mean, whereas repeated buffered spatial validation supported only grouped 3D predominance, not stable dominance of an individual variable. Protecting mature canopies, pedestrian-visible greenery, and multilayer vegetation may therefore be evaluated locally, with thermal performance verified by site-specific measurements because the LL models showed limited spatial transferability.

### 3.5. Limitations and Directions for Future Research

This study is geographically and temporally bounded. The analysis was confined to the central urban area of Wuhan and used mean summer LST derived from June–August 2022. The 2020–2024 ERA5-Land air-temperature and relative-humidity data provide recent regional climate context only and do not constitute multi-year validation of the vegetation–LST relationships. Although the findings may be informative for cities with comparable density and summer heat conditions, they should not be assumed to transfer to different climates, urban forms, seasons, or years. Buffered spatial CV evaluated held-out locations within Wuhan, not external cities or future periods. Multi-city and multi-year analyses are therefore required to assess the geographic and temporal generalizability of the identified relationships. Specifically, future validation should prioritize comparative multi-city research across contrasting climates, urban forms, vegetation compositions, and development densities.

The thermal and vegetation measurements also have interpretive limits. LST_mean effectively represents surface thermal conditions and their spatial variation, but it does not directly measure near-surface air temperature, mean radiant temperature, or pedestrian thermal comfort. Remotely sensed LST may differ from beneath-canopy and pedestrian-level conditions [33], while field-based street-tree studies commonly combine air temperature, humidity, wind speed, and radiation to evaluate indices such as physiological equivalent temperature (PET) or UTCI [5]. In addition, the available vegetation datasets did not include species identity or detailed functional traits such as evergreen–deciduous status, leaf characteristics, canopy porosity, stomatal behavior, or water-use strategy. Because these characteristics may modify shading and transpiration beyond what can be represented by GVI, NV_mean, CH_mean, and CH_sd, future studies should integrate species inventories and functional-trait measurements with the present street-scale framework.

Street-view and remotely sensed inputs introduce additional measurement uncertainty. GVI estimates can be sensitive to the vegetation classes included in semantic segmentation, because different class combinations can materially change mapped GVI even when applied to the same imagery [36]. For multi-temporal street-view assessment, image acquisition season and viewing angle can also affect greenery estimates [37]. Landsat-derived LST is likewise conditional on the retrieval algorithm, atmospheric inputs, sensor characteristics, and land-surface emissivity assumptions [38]. These potential uncertainties may propagate into the estimated vegetation–LST relationships, but they were not quantified here as a study-specific bias. Although residual spatial diagnostics, buffered spatial CV, spatial-block bootstrap, and repeated spatial validation were conducted, a systematic sensitivity analysis across alternative sampling intervals, study-unit radii, temporal windows, and selected processing settings was beyond the present scope. Consequently, the stability of the reported nonlinear relationships and zero-crossing ranges under those alternative analytical settings remains incompletely evaluated.

The model specification and spatial design impose further limitations. The predictor set did not systematically include building density and height, sky openness, road width and orientation, surface materials, proximity to water bodies, traffic intensity, human activity, or synchronous meteorological conditions. These omitted factors are established components of street-scale thermal environments [19,39,40] and likely contribute to the modest R^2^ values and remaining residual structure. Moreover, the 150 m study units were generated at 50 m intervals as overlapping sliding windows. This design provides continuous representation along the road network, but neighboring units are not statistically independent, so the nominal sample size should not be interpreted as an equivalent number of independent observations. The significant OLS residual Moran’s I confirms that spatial dependence remained after accounting for the seven plant-form variables. Buffered spatial CV, spatial-block bootstrap resampling, and repeated spatial validation reduce the risk of overstating predictive robustness, but they do not eliminate all dependence or establish causal inference. Conventional OLS standard errors and *p*-values should therefore be interpreted as within-sample associations rather than spatially robust hypothesis tests. Similarly, SHAP importance and zero-crossing ranges are conditional on the fitted RF models, the selected predictors, and correlations among them; they do not identify physical mechanisms, ecological thresholds, or universal planning standards. Because the HH and LL zones were identified from the spatial configuration of the same LST surface subsequently represented by LST_mean, the zone-specific models are conditional analyses of outcome-defined subsets. The overall, HH, and LL groups are not statistically independent, and between-zone differences in coefficients or SHAP importance should not be interpreted as independent evidence of thermal-regime effect modification. Future work should integrate the omitted urban and meteorological covariates, test spatial error or lag models and spatially robust covariance estimators, and use multi-period field measurements or quasi-experimental designs to evaluate whether the reported associations persist under more independent and causally informative conditions.

The dense 50 m sampling and overlapping 150 m sliding-window design was computationally feasible for the present study and provided continuous street-scale coverage; overlap is an intended feature of this framework rather than a sampling error. Nevertheless, spatial aggregation for 42,603 study units, distance-based diagnostics, repeated RF fitting, and SHAP calculations increase data-processing and model-interpretation costs. Scaling the workflow to larger metropolitan regions, multiple cities, or finer spatial and temporal resolutions will therefore require explicit computational benchmarking and more efficient implementation. Urban digital twin research similarly identifies data integration and quality, model complexity and uncertainty, computational resources, spatial and temporal resolution, and validation as linked implementation challenges [41]. Thus, scalability warrants further evaluation even though the current workflow is feasible.

Future research should combine longitudinal street-view and thermal monitoring to test whether the vegetation–LST relationships, nonlinear responses, and model-derived zero-crossing ranges remain stable across seasons and years [31,37]. Urban digital twin frameworks could then integrate dynamic environmental observations with 2D and 3D urban representations for scenario visualization and decision support [41]. Finally, coupling the RF–SHAP relationships with process-based street-canyon or microscale climate simulations could examine the airflow, shading, radiation, and transpiration processes underlying the statistical patterns rather than replacing machine learning [42].

Future model assessment should also compare RF with XGBoost, LightGBM, CatBoost, Gaussian process regression, and graph-based neural networks under identical predictor sets, spatial folds, tuning budgets, and the same 300 m buffered exclusion rule. Without a common spatial-validation framework, differences in random-split accuracy would not provide a fair test of algorithmic generalization.

## 4. Materials and Methods

### 4.1. Study Area

This study focuses on the central urban area of Wuhan, situated in central China along the middle reaches of the Yangtze River. Wuhan is a prominent megacity within the Yangtze Economic Belt, distinguished by rapid urbanization, high-density development, a complex hydrological network, and hot, humid summer conditions. The accelerated urban expansion has significantly transformed the surface cover composition, thereby influencing the spatial distribution of urban surface thermal characteristics [3]. The 2020–2024 ERA5-Land climatology shows a pronounced seasonal cycle in Wuhan. From January to December, mean monthly 2 m air temperatures across the Wuhan administrative area were 5.5, 7.8, 13.7, 18.0, 22.4, 26.4, 28.6, 29.4, 25.6, 18.4, 13.5, and 6.0 °C, respectively. The corresponding monthly relative humidity values were 71.9%, 74.5%, 73.9%, 73.2%, 75.9%, 79.7%, 81.3%, 74.8%, 70.3%, 69.8%, 69.1%, and 63.5%. During June–August, the three five-year monthly mean temperatures averaged 28.1 °C, while relative humidity averaged 78.6%, characterizing the recent hot and humid summer climate context within which the June–August 2022 LST analysis is situated. These ERA5-Land values describe multi-year near-surface air temperature and relative humidity and are distinct from the Landsat-derived mean summer LST for June–August 2022 used as the dependent variable.

Previous research indicates that elevated LST zones in Wuhan are concentrated in the central urban core, industrial areas, and intensely built-up areas, whereas water bodies and vegetated areas generally show lower LST [3]. This climatic and urban context makes Wuhan’s central urban area suitable for examining how street-scale 2D planar morphology and 3D vertical vegetation structure are associated with LST and for informing targeted street-greening and heat-mitigation strategies [27].

### 4.2. Data Sources and Preprocessing

This study integrates datasets encompassing LST, GVI, green-space distribution, 3D green volume, and canopy height to establish a street-scale database linking vegetation morphology with the thermal environment. The study area boundary was delineated in accordance with the central urban area defined by the Wuhan Territorial Spatial Master Plan (2021–2035), with the Third Ring Road serving as the principal spatial delimiter (Figure 1A). To maintain consistency in measurements of distance, area, buffer zones, and patch morphology, all spatial data were projected using the Universal Transverse Mercator coordinate system, Zone 50N (UTM 50N). Sampling points were generated along the processed road network (Figure 1B), and 150 m-radius street-scale study units were constructed around these points (Figure 1C). Variables including LST_mean, GVI, NV_mean, CH_mean, CH_sd, A, P_mean, and P_A were aggregated at the scale of these study units. These variables were subsequently employed in correlation analyses, linear regression, random forest modeling, and SHAP interpretation. The ERA5-Land air-temperature and relative-humidity data were used exclusively to characterize the regional climate background and were not incorporated into the street-scale variable database or any statistical model. The fundamental attributes of the datasets utilized are summarized in Table 4.

#### 4.2.1. Land Surface Temperature

LST data were acquired from the Landsat surface-temperature product available on the Google Earth Engine (GEE) platform. The analysis used valid observations from June through August 2022, corresponding to the summer period examined in this study. This period was selected to characterize summer surface-temperature variation at the street scale within Wuhan’s central urban area and does not represent a long-term climatic normal. The product’s approximately 30 m spatial resolution supports local aggregation within the 150 m-radius study units.

To ensure data quality, pixels affected by clouds, cloud shadows, and other invalid observations were excluded using the product quality-assessment bands. Following unit conversion, all valid June–August LST observations were averaged on a pixelwise basis to generate the 2022 mean summer LST raster, which was subsequently clipped to Wuhan’s central urban area and projected to WGS 84/UTM Zone 50N (EPSG:32650). For each 150 m-radius street-scale study unit, the mean of all valid LST pixels within the unit was calculated and designated as the dependent variable, LST_mean. This common spatial aggregation enabled LST_mean to be matched with the seven plant-form variables within the same analytical units.

#### 4.2.2. Road Network and Study Units

Road network data were obtained from OpenStreetMap (OSM). Major, secondary, and branch roads within Wuhan’s central urban area were cleaned and simplified to single centerlines. Topology checks, duplicate-line removal, and centerline simplification reduced redundant sampling caused by separated carriageways, parallel auxiliary roads, duplicated segments, and fragmented features. Sampling points were then generated at 50 m intervals along the processed centerlines for street-view image acquisition, study-unit delineation, and variable extraction. Road-based sampling has been widely used to represent pedestrian-view greenery and near-ground street conditions [17,27,44].

Using the processed street-centerline network, sampling points were placed at 50 m intervals to provide dense and continuous coverage of street environments and to capture short-distance variations in roadside vegetation and thermal conditions. Circular buffers with a radius of 150 m were then delineated around the sampling points to form street-scale study units. The selected radius represents a compromise between local street specificity and the stability required for multi-source data aggregation. At this scale, each study unit characterizes the surrounding plant morphology and thermal environment of a street location while remaining sufficiently localized to trace spatial variation along the road network. Because the Landsat LST data have a spatial resolution of 30 m, each study unit contains multiple LST pixels, allowing LST_mean to be calculated from a local neighborhood rather than from a single pixel and thereby reducing sensitivity to pixel-level variation. The same spatial extent also contains sufficient green-space patches and canopy-height observations for the joint calculation of A, P_mean, P_A, NV_mean, CH_mean, and CH_sd on a common analytical basis. Previous street-level thermal studies have similarly used buffer-based extraction around street-view sampling points and have demonstrated that model results may be sensitive to the selected buffer radius [17,44].

Because the 150 m radius is larger than the 50 m sampling interval, adjacent study units overlap substantially. This overlap is intentional and reflects a moving-window design for continuous road networks rather than a set of mutually exclusive spatial zones. Each sampling point serves as the center of a local 150 m analytical window, whereas the 50 m interval ensures high-density sampling and spatial continuity along street corridors. The overlapping study units therefore capture gradual changes in plant morphology and LST along the road network. At the same time, their shared spatial coverage may introduce dependence among neighboring observations; accordingly, the study units were not interpreted as fully independent samples.

Following the establishment of sampling points and their corresponding buffers, spatial filtering was applied using the boundary of Wuhan’s central urban area. Sampling points whose centers were located within the study area, together with their associated study units, were retained. Of 42,604 candidate records, one invalid record was excluded during data cleaning, yielding 42,603 valid street-scale study units for all subsequent analyses.

To evaluate this potential spatial dependence empirically, the spatial autocorrelation of the OLS residuals was subsequently examined using Global Moran’s I at distance thresholds of 150, 300, and 500 m, as described in Section 4.3.1.

#### 4.2.3. Plant-Form Variables

The seven plant-form variables comprise 2D metrics (A, P_mean, and P_A) and 3D metrics (GVI, NV_mean, CH_mean, and CH_sd). The 2D metrics were derived from Gaofen-2 (GF-2) green-space vector data used in prior research [43]. These metrics are established measures in studies of green-space configuration and LST [6,10,12]. Within each street-scale study unit, A is the total green-space area, P_mean is the mean patch perimeter, and P_A is the perimeter–area ratio of green space. When a study unit contained no green-space patches, A, P_mean, and P_A were assigned zero.

The GVI was extracted from Baidu Street View images to quantify visible greenery from a pedestrian viewpoint. Using the road network of Wuhan’s central urban area, sampling points were generated at 50 m intervals, and multi-directional street-view images were acquired through the Baidu Map Application Programming Interface (API). Six images were captured at each sampling location at azimuth angles of 0°, 60°, 120°, 180°, 240°, and 300°. Prior studies have commonly used multi-directional street-view imagery and semantic segmentation to quantify near-ground greenery and streetscape conditions from the pedestrian perspective [18,27]. In the present study, a semantic segmentation model classified each image at the pixel level, and GVI was calculated as the ratio of vegetation pixels to total pixels. The six directional GVI values were averaged for each sampling point, and this point-level mean was assigned to the corresponding study unit centered at the same location.

NV_mean was derived from the Wuhan urban 3D greenery dataset. Following the method described in Chen et al. [43], Landsat-8 imagery was first used to estimate LAI through a parametric regression model combining spectral band reflectance and the NDVI. The estimated LAI was then used to calculate 3D green volume, and the mean value within each 150 m-radius study unit was extracted as NV_mean to represent the mean 3D green volume within the study unit. Canopy-height data were obtained from the 1 m global tree/canopy height product released by Meta Sustainability and World Resources Institute (Meta/WRI), and were used to characterize the vertical structure of vegetation around streets. Recent 2D/3D urban morphology studies have also used the 1 m Meta/WRI canopy-height product together with high-resolution green-space data to characterize vegetation vertical structure for machine-learning-based LST modeling [21]. After spatial harmonization, the canopy-height data were summarized within each 150 m-radius study unit to calculate CH_mean and CH_sd. CH_mean represents the overall canopy-height level within the study unit, while CH_sd represents canopy-height variation and vertical structural heterogeneity. Previous studies have shown that tree height, canopy quality, and vertical structure can influence shading, radiation interception, and thermal regulation processes; meanwhile, the relationship between tree height and LST may also be moderated by surrounding building height, suggesting that 2D green-space area alone cannot fully explain differences in vegetation cooling effects [7,19]. Finally, all plant-form variables were summarized for the 42,603 valid street-scale study units and spatially matched with LST_mean, forming the unified database used for correlation analysis, linear regression, random forest modeling, and SHAP interpretation.

### 4.3. Model Development and Interpretation Framework

**Figure 10:**
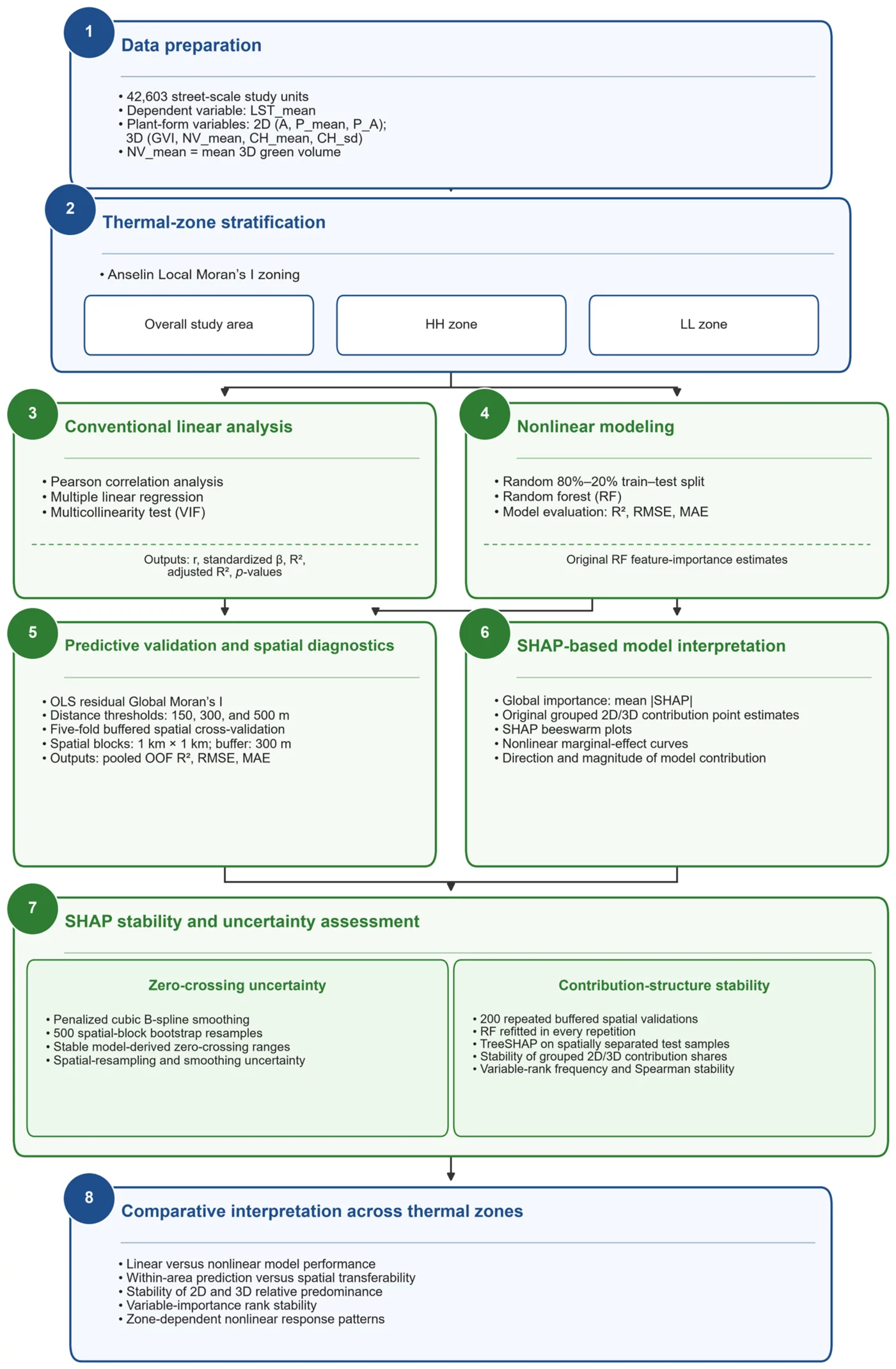
Workflow of data preparation, thermal-zone stratification, model development, predictive validation, spatial diagnostics, SHAP interpretation, and stability assessment. Street-scale study units were analyzed for the overall study area, the HH zone, and the LL zone identified using Anselin Local Moran’s I. Conventional linear analysis included Pearson correlation, ordinary least squares regression, and variance inflation factor diagnostics, whereas nonlinear modeling was conducted using random forest. Predictive validation incorporated the original random 80–20% train–test split and five-fold buffered spatial cross-validation, together with Global Moran’s I analysis of OLS residuals. SHAP-based interpretation included global importance, grouped 2D/3D contribution point estimates, beeswarm plots, and nonlinear marginal-effect curves. Stability assessment comprised 500 spatial-block bootstrap resamples for model-derived zero-crossing ranges and 200 repeated buffered spatial validations in which RF models were refitted and TreeSHAP values were calculated for spatially separated test samples to evaluate grouped contribution shares and variable-rank stability.

The 42,603 valid street-scale study units formed three analytical groups: the overall study area, the HH zone, and the LL zone. Pearson correlation and full-sample OLS characterized bivariate and multivariable linear associations, and VIF assessed multicollinearity. LR and RF then used identical random training and testing partitions, with performance evaluated by R^2^, RMSE, and MAE. SHAP analysis of the RF models quantified global importance, contribution direction, and nonlinear marginal relationships for GVI, NV_mean, CH_mean, CH_sd, A, P_mean, and P_A. Figure 10 summarizes eight linked stages: data preparation, thermal-zone stratification, conventional linear analysis, nonlinear modeling, predictive validation and spatial diagnostics, SHAP-based interpretation, SHAP stability and uncertainty assessment, and comparative interpretation across analytical groups. Validation distinguished within-area performance from spatially separated prediction, while stability analyses examined zero-crossing uncertainty, grouped contribution shares, and variable ranks.

Local thermal zones were identified using the Anselin Local Moran’s I tool in ArcGIS Pro 3.5.0 (Esri, Redlands, CA, USA). The input feature class, Wuhan_LST_Polygon2, was generated by polygonizing the integerized 30 m mean-summer LST raster, so gridcode retained integerized LST values rather than feature identifiers. Spatial relationships were conceptualized using inverse-distance weighting based on Euclidean distance, with a 90 m threshold corresponding to three source-raster cells and row standardization of the spatial weights. Statistical significance was evaluated using 999 random permutations, and false discovery rate correction was not applied. Under this configuration, polygons with pseudo *p*-values below 0.05 were classified in the COType field as statistically significant zones or spatial outliers at the 95% confidence level. Polygons characterized by high LST values surrounded by similarly high values were classified as High–High (HH) zones, whereas polygons characterized by low values surrounded by similarly low values were classified as Low–Low (LL) zones. High–Low (HL) and Low–High (LH) categories represented spatial outliers and were not included in the subsequent zone-specific analyses. The source-data audit identified 83,680 LST polygons, including 16,713 HH polygons, 17,591 LL polygons, and 3358 zero-neighbor polygons coded as NN. Street-scale study units were assigned to the HH or LL zone according to whether their center points were located within the corresponding polygons. An independent point-in-polygon verification exactly reproduced the 5029 HH and 829 LL study units, with no duplicate PointID values. Therefore, the HH and LL zones represent statistically significant local spatial zones rather than groups defined using predetermined LST thresholds.

#### 4.3.1. Conventional Linear Models

To elucidate the fundamental linear associations between plant-form variables and LST_mean, this study initially computed Pearson correlation coefficients. Subsequently, multiple linear regression models were developed independently for the overall study area, the HH zone, and the LL zone. In these models, LST_mean served as the dependent variable, while the explanatory variables included GVI, NV_mean, CH_mean, CH_sd, A, P_mean, and P_A. VIF values were calculated separately for the overall study area, the HH zone, and the LL zone using the complete samples included in the corresponding full-sample OLS models. The diagnostic was conducted for the seven explanatory variables, namely GVI, NV_mean, CH_mean, CH_sd, A, P_mean, and P_A, with LST_mean excluded. A VIF value below 5 was used to indicate the absence of severe multicollinearity.

Spatial autocorrelation in the OLS residuals was further evaluated using Global Moran’s I to determine whether unexplained LST variation remained spatially structured after accounting for the seven plant-form variables. The residuals were linked to the center coordinates of the street-scale study units and projected to WGS 84/UTM Zone 50N. Binary distance-band spatial weights with row standardization were constructed at thresholds of 150, 300, and 500 m. The 150 m threshold represented the immediate neighborhood scale corresponding to the radius of the study units, whereas the 300 and 500 m thresholds assessed sensitivity to broader neighborhood definitions. Global Moran’s I was calculated separately for the overall study area, the HH zone, and the LL zone. Study units without neighbors at a given threshold were retained in the analytical dataset, and zero-neighbor counts were recorded separately for each analytical group and threshold. The expectation and variance of Global Moran’s I were evaluated using the analytical randomization variance, and two-sided *p*-values were obtained from the normal approximation to the corresponding z-scores. Values below 0.001 were reported as *p* < 0.001 rather than *p* = 0. When significant residual spatial autocorrelation was detected, conventional OLS coefficient *p*-values were interpreted as nominal descriptive statistics rather than spatially robust inference.

The full-sample OLS models described above were used to summarize coefficient estimates, VIF diagnostics, and residual spatial autocorrelation. For predictive comparison with RF, separate LR models were fitted under a random 80–20% train–test split using the same dependent and explanatory variables. The split produced training and testing sample sizes of 34,082 and 8521 for the overall study area, 4023 and 1006 for the HH zone, and 663 and 166 for the LL zone. LR performance on the held-out testing samples was evaluated using R^2^, RMSE, and MAE and was compared directly with RF performance under the identical partition.

#### 4.3.2. Nonlinear Modeling and Spatial Validation

Because plant-form variables may have nonlinear associations with LST_mean, including diminishing marginal responses, non-monotonicity, and directional changes, RF was used to complement LR. RF can represent nonlinear relationships and interactions without a predetermined functional form and is widely used in urban thermal research [20,26]. Beyond regression, RF is also applicable to remote-sensing image classification. In a comparison of four algorithms for land-cover classification in a gold-mining area, Oo et al. reported that RF achieved the highest overall accuracy (95.85%), outperforming support vector machine, classification and regression trees, and maximum-likelihood classification [45]. Here it was used to examine range-dependent model relationships, not causal effects. For the *i*-th street-scale study unit, the RF prediction was expressed as the average output of the regression trees following Breiman’s formulation [28], as shown in Equation (1):

*ŷ_i_* = (1/*T*) Σ_*t*=1_*^T^*
*h_t_*(*X_i_*) (1)

where *ŷ_i_* is the predicted LST_mean of the *i*-th study unit, *T* is the number of regression trees in the random forest, and *h_t_*(*X_i_*) represents the prediction of the *t*-th regression tree based on the input variables *X_i_*. The input variables included GVI, NV_mean, CH_mean, CH_sd, A, P_mean, and P_A.

All RF models were implemented with 300 regression trees, a maximum tree depth of 16, a minimum of five samples per leaf, sqrt feature sampling at each split, and random_state = 20260616. The remaining RandomForestRegressor parameters were retained at their scikit-learn defaults. The original 80–20% split used test_size = 0.20 and random_state = 20260616. The same fixed RF hyperparameters were used for the overall study area, the HH zone, and the LL zone, as well as for the random holdout analysis, five-fold buffered spatial cross-validation, and repeated buffered spatial validation. No testing sample was used for hyperparameter tuning. Within each analytical group, LR and RF used identical dependent and explanatory variables and the same training–testing partition. Random-holdout performance was assessed on the held-out testing sample, whereas spatial cross-validation performance was evaluated from pooled out-of-fold predictions as described below.

Conventional RF importance summarizes contribution magnitude but not contribution direction or marginal relationships across feature ranges. SHAP analysis was therefore used to interpret the fitted RF models more comprehensively [20,26].

To evaluate the spatial robustness and transferability of model performance, five-fold buffered spatial block cross-validation was conducted. Study-unit centroids were projected to WGS 84/UTM Zone 50N and assigned to 1 km × 1 km spatial blocks. Complete blocks were allocated using GroupKFold with *n*_splits = 5. For each fold, all study units in the selected blocks were retained as the test set, whereas candidate training units within 300 m of any test centroid were excluded to prevent direct overlap between 150 m-radius study units. LR and RF used identical test folds and explanatory variables, and RF retained the fixed hyperparameters described above. Pooled out-of-fold R^2^, RMSE, and MAE were reported; negative R^2^ values were retained, and no test data were used for model selection or tuning.

No universal cutoff was imposed to classify R^2^, RMSE, or MAE as acceptable. These metrics were interpreted comparatively for LR and RF evaluated under the same data partition and validation framework, with higher R^2^ and lower RMSE and MAE indicating relatively stronger predictive performance rather than compliance with a universal goodness criterion.

#### 4.3.3. SHAP Model Interpretation and Zero-Crossing Analysis

SHAP decomposes each RF prediction into contributions from the input variables, enabling assessment of global importance, contribution direction, and nonlinear response patterns. For the *i*-th street-scale study unit, the model prediction is represented by Equation (2) [29]:

f(*X_i_*) = *φ*_0_ + Σ_*k*=1_^*p*^
*φ*_*i*,*k*_ (2)

where f(*X_i_*) is the model prediction for the *i*-th street-scale study unit, *φ*_0_ is the baseline prediction, *φ*_*i*,*k*_ is the SHAP contribution of the *k*-th variable to that prediction, and *p* is the number of input variables. A positive SHAP value increases RF-predicted LST_mean relative to the model baseline, whereas a negative value decreases it. These are model contributions, not causal thermal effects. A larger absolute SHAP value indicates a larger contribution magnitude to the model output.

Global importance was measured as the mean absolute SHAP value of each variable, as shown in Equation (3) [46]:

*I_k_* = (1/*n*) Σ_*i*=1_^*n*^ |*φ*_*i*,*k*_| (3)

where *I_k_* represents the global importance of the *k*-th plant-form variable, *n* is the sample size, and *φ*_*i*,*k*_ is the SHAP value of the *k*-th variable for the *i*-th sample. A larger *I_k_* indicates a greater overall contribution of that variable in the random forest model.

For the overall study area, the HH zone, and the LL zone, mean absolute SHAP values summarized the global contributions of GVI, NV_mean, CH_mean, CH_sd, A, P_mean, and P_A. Beeswarm plots then showed how lower and higher feature values corresponded to positive or negative contributions to RF-predicted LST_mean.

To characterize nonlinear response patterns, exact TreeSHAP values were calculated for all valid study units in each analytical group using the RF model fitted under the original 80–20% train–test split. For each group–variable combination, the relationship between the original variable value and its corresponding SHAP value was fitted using a penalized cubic B-spline with a second-difference penalty. Ten internal knots were positioned according to the empirical feature distribution, and the smoothing parameter was selected by generalized cross-validation from a predefined logarithmic grid. Curve fitting and zero-crossing detection were restricted to the 2.5th–97.5th percentile range of each variable, and predictions were generated on a 500-point grid. A zero crossing was identified where the fitted response intersected SHAP = 0, with the direction recorded as either positive-to-negative or negative-to-positive.

The spatial uncertainty of the identified crossings was evaluated through 500 spatial-block bootstrap resamples. Study units were grouped into 1 km × 1 km spatial blocks in WGS 84/UTM Zone 50N, and complete blocks were sampled with replacement in each replicate. The smoothing parameter, fitted response curve, and zero crossings were recalculated for every bootstrap sample. Bootstrap crossings were matched one-to-one with the corresponding full-data candidates, after which the median, 2.5th percentile, 97.5th percentile, detection rate, and direction-consistency rate were calculated. A candidate was retained as a stable model-derived zero-crossing range when its detection rate was at least 70%, its direction-consistency rate was at least 80%, its 95% interval occupied no more than 50% of the effective search range, and its bootstrap distribution was not multimodal. These ranges quantify spatial-resampling and smoothing uncertainty conditional on the fitted RF–SHAP models and should not be interpreted as causal confidence intervals, ecological thresholds, or universally applicable planning control criteria.

#### 4.3.4. Spatial Stability Assessment of SHAP Contributions

To evaluate whether the relative SHAP contribution structure remained stable under alternative spatial partitions, repeated buffered spatial validation was conducted separately for the overall study area, the HH zone, and the LL zone. Study-unit centroids were projected to WGS 84/UTM Zone 50N and assigned to a common 1 km × 1 km spatial grid. For each analytical group, 200 valid repetitions were generated. In each repetition, complete spatial blocks containing approximately 20% of the study units were selected as the test sample, while candidate training units located within 300 m of any testing unit were excluded to reduce direct spatial overlap between the 150 m-radius study units. The RF model was refitted using the same hyperparameters in every repetition, and exact TreeSHAP values were calculated only for the spatially separated test sample.

For each repetition, the mean absolute SHAP values of A, P_mean, and P_A were summed to represent the aggregated 2D contribution, whereas those of GVI, NV_mean, CH_mean, and CH_sd were summed to represent the aggregated 3D contribution. Each group total was divided by the combined 2D and 3D total to obtain the aggregated mean absolute SHAP contribution share. The stability of the grouped contribution structure was summarized using the median and empirical 2.5th–97.5th percentiles across the 200 repetitions. A relative predominance was considered stable only when the corresponding percentile range remained entirely above the 50% reference.

Variable-level stability was further assessed using rank-frequency distributions and Spearman rank correlations between the variable-importance rankings obtained in each repetition and the original RF ranking. These analyses were used to distinguish stable group-level contribution patterns from possible redistribution of SHAP importance among individual predictors. The resulting percentile ranges and rank frequencies characterize sensitivity to spatial resampling and should not be interpreted as causal confidence intervals or evidence of stable spatial transferability.

The final reproducibility analyses were conducted in Python 3.12.13 using NumPy 2.4.6, pandas 3.0.3, scikit-learn 1.9.0, SHAP 0.52.0, and pyproj 3.7.2. Spatial coordinates were processed in WGS 84/UTM Zone 50N (EPSG:32650). 

## 5. Conclusions

This study established a continuous street-scale analytical framework for examining associations between plant morphology and LST across 42,603 study units in the central urban area of Wuhan. Study units were defined at 50 m intervals along the road network using a 150 m radius, enabling 2D planar metrics (A, P_mean, and P_A) and 3D structural metrics (GVI, NV_mean, CH_mean, and CH_sd) to be evaluated on common spatial support. The overall study area and the Anselin Local Moran’s I-derived HH and LL zones were compared using LR, RF, SHAP, residual spatial diagnostics, buffered spatial cross-validation, repeated spatial validation, and spatial-block bootstrap analysis.

Model performance depended strongly on the validation design. Under the random 80–20% split, RF achieved higher testing R^2^ than LR in the overall study area (0.4002 versus 0.2153), the HH zone (0.3539 versus 0.1940), and the LL zone (0.4853 versus 0.0488). After five-fold buffered spatial cross-validation, RF retained only a modest advantage overall (0.2237 versus 0.1940), LR exceeded RF in the HH zone (0.1427 versus 0.0965), and both models produced negative pooled out-of-fold R^2^ in the LL zone (−0.0942 for LR and −0.0231 for RF). Thus, the nonlinear structure captured by RF improved prediction within the randomly partitioned sample but did not provide a consistent advantage for spatially separated observations. The negative LL values indicate performance below the constant-mean benchmark calculated from the pooled out-of-fold observations, not model failure in the original within-area analysis.

The SHAP results likewise require a distinction between original-model point estimates and spatial stability. In the original fitted RF models, A, P_mean, and NV_mean had the largest mean absolute SHAP contributions overall; A, P_A, and P_mean ranked highest in the HH zone; and GVI, CH_sd, and CH_mean ranked highest in the LL zone. The original overall grouped contribution was near balanced (52.5% for 2D and 47.5% for 3D), and repeated validation yielded a median 2D share of 52.1% with a 46.5–58.8% range that crossed 50%; stable overall 2D predominance was therefore not supported. By contrast, repeated spatial validation supported stable 2D relative predominance in the HH zone (61.0% [55.5–66.7%]) and stable 3D relative predominance at the grouped level in the LL zone (61.9% [52.3–70.3%]). The LL individual-variable rankings remained unstable, so no single variable can be regarded as consistently predominant there. Nonlinear SHAP relationships and multiple directional changes were widespread, and 39 of 40 full-sample zero-crossing candidates met the predefined stability criteria. These crossings describe model-conditional directional transitions in contributions to RF predictions; they are not ecological thresholds, explained-variance shares, causal effects, or universal planning criteria.

These findings indicate that street-greening assessment should consider both 2D configuration and 3D structure and should adapt their relative emphasis to local thermal context rather than relying on a single universally preferred indicator. They also show the value of spatial validation when machine-learning models are used to assess urban thermal environments. Nevertheless, inference is limited to Wuhan’s climatic and urban context, summer 2022 LST, the current 50 m sampling interval and 150 m-radius study-unit definition, remaining spatial dependence, and restricted spatial generalizability. The 3D variables are structural proxies and do not directly capture species composition, evergreen–deciduous status, leaf traits, leaf area index or density, canopy porosity, or physiological processes. Future multi-city and multi-season validation with broader vegetation, built-environment, and meteorological information is needed before the model-derived patterns can support more transferable planning guidance.

## Figures and Tables

**Table 1:** Descriptive statistics of LST and plant-form variables across the overall study area and the HH and LL zones.

Variable	Unit	Overall Study Area (*n* = 42,603)	HH Zone (*n* = 5029)	LL Zone (*n* = 829)
LST_mean	°C	46.46 ± 2.90	49.74 ± 1.94	39.50 ± 2.45
GVI	%	23.66 ± 17.78	19.11 ± 16.64	30.37 ± 21.77
NV_mean	index	1.79 ± 0.19	1.73 ± 0.15	1.82 ± 0.27
CH_mean	m	1.28 ± 2.14	0.79 ± 1.30	1.87 ± 2.56
CH_sd	m	2.16 ± 1.50	1.79 ± 1.27	2.65 ± 1.87
A	m^2^	19,649.46 ± 12,673.02	13,469.56 ± 9827.96	24,940.11 ± 15,615.58
P_mean	m	40.89 ± 23.64	37.14 ± 19.18	56.19 ± 46.59
P_A	m^−1^	0.0045 ± 0.0437	0.0057 ± 0.0261	0.0141 ± 0.1119

Note: Values are reported as mean ± standard deviation.

**Table 2:** Stability of aggregated mean absolute SHAP contribution shares under repeated buffered spatial validation.

Analytical Group	Original 2D Share (%)	Repeated 2D Share, Median [2.5th–97.5th] (%)	Original 3D Share (%)	Repeated 3D Share, Median [2.5th–97.5th] (%)	Interpretation
Overall study area	52.5	52.1 [46.5–58.8]	47.5	47.9 [41.2–53.5]	Sensitive to spatial resampling
HH zone	60.8	61.0 [55.5–66.7]	39.2	39.0 [33.3–44.5]	Stable 2D relative predominance
LL zone	31.5	38.1 [29.7–47.7]	68.5	61.9 [52.3–70.3]	Stable 3D relative predominance

Note: Original values are point estimates obtained from the original RF models. Repeated estimates were derived from 200 valid buffered spatial splits for each analytical group. In every repetition, the RF model was refitted and exact TreeSHAP values were calculated only for the spatially separated test sample. Complete 1 km × 1 km spatial blocks were assigned to the test set, and candidate training units within 300 m of testing units were excluded. Percentile ranges represent empirical 2.5th–97.5th percentiles across the 200 repetitions and should not be interpreted as causal confidence intervals or explained-variance shares.

**Table 3:** Model-derived SHAP zero-crossing estimates and spatial-block bootstrap 95% intervals across the overall study area and the HH and LL zones.

Variable	Overall Study Area	HH Zone	LL Zone
A (m^2^)	19,455 [19,147–19,786], Pos → Neg	14,213 [13,941–14,507], Pos → Neg	7161 [4686–8701], Neg → Pos; 29,194 [27,943–30,278], Pos → Neg
P_mean (m)	37.22 [37.00–37.42], Pos → Neg	37.75 [37.45–38.08], Pos → Neg	25.19 [24.35–42.74], Pos → Neg; 29.73 [28.44–42.09], Neg → Pos; 43.64 [42.69–52.20], Pos → Neg
P_A (m^−1^)	0.002342 [0.002303–0.002386], Neg → Pos	0.003494 [0.003413–0.003556], Neg → Pos	0.000998 [0.000888–0.001140], Neg → Pos; 0.007098 [0.006529–0.007800], Pos → Neg
GVI (%)	13.94 [13.52–14.35], Pos → Neg	0.93 [0.83–1.06], Pos → Neg; 31.92 [31.24–32.51], Neg → Pos	3.21 [2.81–4.25], Neg → Pos; 69.75 [68.84–71.09], Pos → Neg
NV_mean (index)	1.551 [1.541–1.559], Neg → Pos; 1.905 [1.895–1.913], Pos → Neg	1.546 [1.539–1.553], Neg → Pos; 1.612 [1.607–1.617], Pos → Neg; 1.659 [1.652–1.666], Neg → Pos; 1.964 [1.947–1.982], Pos → Neg	1.720 [1.710–1.731], Neg → Pos; 1.931 [1.920–1.952], Pos → Neg; 2.056 [2.022–2.069], Neg → Pos
CH_mean (m)	0.085 [0.070–0.098], Neg → Pos; 0.869 [0.852–0.886], Pos → Neg	0.155 [0.106–0.168], Neg → Pos; 1.108 [0.968–1.313], Pos → Neg	0.075 [0.045–0.117], Neg → Pos; 1.891 [1.832–1.945], Pos → Neg
CH_sd (m)	1.923 [1.889–1.970], Neg → Pos	0.776 [0.746–0.833], Neg → Pos; 0.998 [0.949–1.045], Pos → Neg; 2.322 [2.223–2.456], Neg → Pos	0.434 [0.279–0.577], Neg → Pos; 2.498 [2.389–2.975], Pos → Neg

Note: Values are reported as the spatial-block bootstrap median [2.5th–97.5th percentile interval]. Pos → Neg and Neg → Pos indicate positive-to-negative and negative-to-positive changes in SHAP contributions, respectively. Zero crossings were searched within the central 95% of each variable distribution and were considered stable when the detection rate was ≥70%, direction consistency was ≥80%, the interval width was ≤50% of the search range, and the crossing distribution was not multimodal. One unstable low-value CH_mean candidate in the overall study area was excluded. The overlapping P_mean intervals in the LL zone represent a broad multistage transition rather than discrete thresholds. These model-conditional ranges are not causal effects, ecological thresholds, or universally transferable planning standards.

**Table 4:** Attributes of the datasets used for model construction and regional climate-context characterization.

Dataset	Variable/Use	Resolution	Time	Source
Landsat-derived LST data	LST_mean	30 m	June–August 2022	Google Earth Engine (Google LLC, Mountain View, CA, USA; no fixed end-user version)
OSM road network	Road sampling points and 150 m-radius study units	/	2022	OpenStreetMap (OSM)
Gaofen-2 (GF-2)-derived green-space vector data	A, P_mean, P_A	1 m	22 October 2022	[43]; original imagery from China Center for Resources Satellite Data and Application
Baidu Street View images	GVI	/	2018–2022	Baidu Maps API (Baidu, Beijing, China); HRNet semantic segmentation
Wuhan urban 3D green volume data	NV_mean	30 m	3 June 2022	Landsat-8 imagery
Meta/WRI global canopy height product	CH_mean, CH_sd	1 m	2020	Meta Sustainability and World Resources Institute (WRI)
ECMWF ERA5-Land climate reanalysis data	Mean monthly 2 m air temperature and relative humidity; regional climate background only	11.1 km	2020–2024	ECMWF ERA5-Land Daily Aggregated, accessed and processed through Google Earth Engine (Google LLC, Mountain View, CA, USA; no fixed end-user version)

## Data Availability

The processed minimal dataset and derived analytical outputs supporting the principal results of this study are provided as Supplementary Dataset S1. The Supplementary Dataset includes the processed street-scale study-unit data, variable definitions, descriptive and linear-analysis results, model-performance results, residual spatial-autocorrelation diagnostics, SHAP importance results, repeated spatial-validation summaries, variable-rank stability results, and model-derived SHAP zero-crossing results. Publicly available source datasets used in this study include Landsat data accessed through Google Earth Engine, OpenStreetMap road-network data, ERA5-Land climate reanalysis data accessed through Google Earth Engine, and the Meta/WRI global canopy-height product. Raw Baidu Street View imagery, GF-2-derived green-space source data, Wuhan urban 3D green volume source data, and other provider-controlled source datasets are not redistributed with this article and remain subject to the access conditions of their respective data providers or data owners.

## Supplementary Materials

The following supporting information can be downloaded at: https://www.mdpi.com/article/10.3390/plants15172561/s1, Supplementary Dataset S1: Processed minimal dataset and derived analytical outputs supporting the principal results of this study (Minimal_Dataset_Zhang_et_al.zip), including 01_study_unit_dataset.csv, 02_variable_dictionary.csv, 03_descriptive_statistics.csv, 04_linear_analysis.csv, 05_model_performance.csv, 06_residual_morans_i.csv, 07_shap_importance_original.csv, 08_repeated_grouped_shap_stability.csv, 09_variable_rank_stability.csv, 10_shap_zero_crossings.csv, 11_zero_crossing_bootstrap_summary.csv, 12_figure_source_summary.xlsx, and README.txt.

## Abbreviations

The following abbreviations are used in this manuscript:

2D	two-dimensional
3D	three-dimensional
A	green-space area
API	Application Programming Interface
CH_mean	mean canopy height
CH_sd	standard deviation of canopy height
CV	cross-validation
GCV	generalized cross-validation
GEE	Google Earth Engine
GF-2	Gaofen-2
GVI	green view index
HH	High–High
HRNet	High-Resolution Network
LAD	leaf area density
LAI	leaf area index
LiDAR	light detection and ranging
LL	Low–Low
LR	linear regression
LST	land surface temperature
LST_mean	mean land surface temperature
MAE	mean absolute error
NDVI	normalized difference vegetation index
NV_mean	mean 3D green volume
OLS	ordinary least squares
OOF	out-of-fold
OSM	OpenStreetMap
P_A	perimeter–area ratio of green space
P_mean	mean patch perimeter
PET	physiological equivalent temperature
R^2^	coefficient of determination
RF	random forest
RMSE	root mean square error
SHAP	SHapley Additive exPlanations
TreeSHAP	SHAP algorithm for tree-based models
UAV	unmanned aerial vehicle
UTCI	Universal Thermal Climate Index
UTM 50N	Universal Transverse Mercator Zone 50N
VIF	variance inflation factor
WGS 84	World Geodetic System 1984
WRI	World Resources Institute
